# Tundra Vegetation Community Type, Not Microclimate, Controls Asynchrony of Above‐ and Below‐Ground Phenology

**DOI:** 10.1111/gcb.70153

**Published:** 2025-04-02

**Authors:** Elise C. Gallois, Isla H. Myers‐Smith, Colleen M. Iversen, Verity G. Salmon, Laura L. Turner, Ruby An, Sarah C. Elmendorf, Courtney G. Collins, Madelaine J. R. Anderson, Amanda Young, Lisa Pilkinton, Gesche Blume‐Werry, Maude Grenier, Geerte Fälthammar‐de Jong, Inge H. J. Althuizen, Casper T. Christiansen, Simone I. Lang, Cassandra Elphinstone, Greg H. R. Henry, Nicola Rammell, Michelle C. Mack, Craig See, Christian Rixen, Robert D. Hollister

**Affiliations:** ^1^ University of Edinburgh Edinburgh UK; ^2^ UK Centre for Ecology and Hydrology Edinburgh Research Station Penicuik UK; ^3^ Department of Forest and Conservation Sciences, Faculty of Forestry University of British Columbia Vancouver British Columbia Canada; ^4^ Environmental Sciences Division Oak Ridge National Laboratory Oak Ridge Tennessee USA; ^5^ School of Geography The University of Nottingham Nottingham UK; ^6^ Princeton University Princeton New Jersey USA; ^7^ Institute of Arctic and Alpine Research, University of Colorado Boulder Colorado USA; ^8^ Department of Ecology and Evolutionary Biology University of Colorado Boulder Colorado USA; ^9^ School of Environmental Sciences Simon Fraser University Burnaby British Columbia Canada; ^10^ Biodiversity Research Centre The University of British Columbia Vancouver British Columbia Canada; ^11^ Département de Biologie Université de Sherbrooke Sherbrooke Quebec Canada; ^12^ Toolik Field Station Institute of Arctic Biology, University of Alaska Fairbanks Fairbanks Alaska USA; ^13^ Department of Ecology and Environmental Science Umeå University Umeå Sweden; ^14^ University of Gothenburg Göteborg Sweden; ^15^ NORCE Norwegian Research Centre AS Bergen Norway; ^16^ Bjerknes Centre for Climate Research Bergen Norway; ^17^ Department of Biology, Terrestrial Ecology Section University of Copenhagen Copenhagen Denmark; ^18^ The University Centre in Svalbard Longyearbyen Norway; ^19^ Department of Botany University of British Columbia Vancouver British Columbia Canada; ^20^ Center for Ecosystem Science and Society Northern Arizona University Flagstaff Arizona USA; ^21^ WSL Institute for Snow and Avalanche Research SLF Davos Switzerland; ^22^ Climate Change, Extremes and Natural Hazards in Alpine Regions Research Centre CERC Davos Dorf Switzerland; ^23^ Grand Valley State University Allendale Michigan USA

**Keywords:** below‐ground, carbon cycling, climate change, permafrost thaw, phenology, root dynamics, root phenology, tundra ecology

## Abstract

The below‐ground growing season often extends beyond the above‐ground growing season in tundra ecosystems and as the climate warms, shifts in growing seasons are expected. However, we do not yet know to what extent, when and where asynchrony in above‐ and below‐ground phenology occurs and whether variation is driven by local vegetation communities or spatial variation in microclimate. Here, we combined above‐ and below‐ground plant phenology metrics to compare the relative timings and magnitudes of leaf and fine‐root growth and senescence across microclimates and plant communities at five sites across the Arctic and alpine tundra biome. We observed asynchronous growth between above‐ and below‐ground plant tissue, with the below‐ground season extending up to 74% (~56 days) beyond the onset of above‐ground leaf senescence. Plant community type, rather than microclimate, was a key factor controlling the timing, productivity, and growth rates of fine roots, with graminoid roots exhibiting a distinct ‘pulse’ of growth later into the growing season than shrub roots. Our findings indicate the potential of vegetation change to influence below‐ground carbon storage as the climate warms and roots remain active in unfrozen soils for longer. Taken together, our findings of increased root growth in soils that remain thawed later into the growing season, in combination with ongoing tundra vegetation change including increased shrub and graminoid abundance, indicate increased below‐ground productivity and altered carbon cycling in the tundra biome.

## Graphical abstract

In tundra ecosystems, plant roots continue growing well beyond the above‐ground growing season productivity peak. We found that plant community type, rather than microclimate, plays a key role in determining root growth patterns, with graminoid roots in particular showing a late‐season growth surge. As the climate warms and tundra vegetation communities shift, these changes could impact long‐term below‐ground carbon storage and overall carbon cycling in arctic and alpine tundra landscapes.

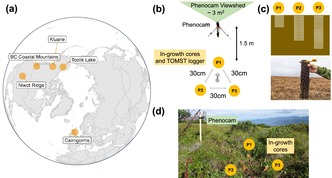

## 1 Introduction

Over the last three decades, many tundra plants have exhibited earlier bud break and growth in response to warmer summer temperatures, and at a rate of change four times higher than for the planet as a whole (Høye et al. 2007; Panchen and Gorelick 2015, 2017; Prevéy et al. 2019; Wookey et al. 1993; Rantanen et al. 2022). Above‐ground (leaf, shoot, flower) phenology varies in timing and in strength of sensitivity to local abiotic drivers (such as snowmelt and surface temperature) and by species (Assmann et al. 2019; Bjorkman et al. 2015; Prevéy et al. 2017). In Arctic Sweden and Western Greenland, the timing of above‐ and below‐ground plant growth has been observed to be asynchronous, with the below‐ground growing season extending up to 50% longer than the above‐ground growing season (Blume‐Werry 2021; Blume‐Werry et al. 2019; Liu et al. 2021; Radville et al. 2016; Sullivan et al. 2007). In addition, below‐ground fine‐root growth has been found to be relatively unresponsive to experimental manipulations of temperature and snowmelt timing (Blume‐Werry et al. 2017). However, previous studies have not tested the asynchrony and drivers of above‐ versus below‐ground root productivity and the timing of root growth across tundra sites and throughout tundra landscapes across microclimates.

Below‐ground plant biomass represents 24% of overall global mean plant biomass, yet in much of the tundra biome approximately 80% of vegetative biomass is found below‐ground (Mokany et al. 2006). Tundra plants have the shallowest roots across all of the world's biomes and are adapted to be highly productive despite the high permafrost table and cold soil conditions (Iversen et al. 2015; Schenk and Jackson 2002; Shaver and Billings 1975). The growth patterns and phenological dynamics of fine roots (narrow‐diameter roots responsible for nutrient and water acquisition) are critically under‐represented in terrestrial ecosystem and carbon models due to the scarcity of data and oversimplification of root‐microenvironment relationships (Smithwick et al. 2014; Warren et al. 2015). Plant roots efficiently convert atmospheric carbon into stable soil carbon (Jones et al. 2009; Sokol and Bradford 2019) and are a large source of decomposable litter, much of which is respired back into the atmosphere (Sullivan et al. 2007; Zona et al. 2022). However, our understanding of the physiological coupling of above‐ and below‐ground phenology and the abiotic drivers of tundra root growth remains limited, hampering our ability to accurately model tundra ecosystem carbon cycling in tandem with climate warming (Smithwick et al. 2014; Warren et al. 2015).

Plant productivity, above‐ground biomass as a whole, and shrub and graminoid abundance are increasing across multiple tundra field sites in concert with climate warming (Berner and Goetz 2022; Bhatt et al. 2013; Elmendorf et al. 2012; Forbes et al. 2010; Myers‐Smith et al. 2011, 2020). Much of this change is attributed to the encroachment and subsequent range expansion of woody shrubs, including increases in both height and extent of individual shrubs and infilling of shrub cover through clonal growth and new recruitment (Forbes et al. 2010; García Criado et al. 2020; Martin et al. 2017; Naito and Cairns 2011; Tape et al. 2006). Graminoid species are also expected to increase in abundance in response to climate change (Bjorkman et al. 2020; Elmendorf et al. 2012) through local phenomena such as flooding or water‐logging via permafrost thaw (Heijmans et al. 2022). While there is ample evidence of tundra ecosystem change based on above‐ground vegetation monitoring, below‐ground biomass and phenology change are far more challenging to track and thus rarely reported (Iversen et al. 2015).

The ways in which roots grow, acquire, and use nutrients, and interact with biotic stimuli vary considerably between plant functional types (de Kroon et al. 2012), and thus any future vegetation range shifts could have important ecological consequences in tundra soils. For example, shrubs often increase root growth earlier in the summer and in shallower soils, while graminoids often root later in summer and in deeper soils near the thaw front (Keuper et al. 2017; McKane et al. 2002; Schwieger et al. 2018; Sullivan et al. 2007). Increased root production in warmer soils could provide more efficient mechanisms of stable sequestration of atmospheric carbon (Sokol and Bradford 2019), but could also lead to greater long‐term losses of soil organic carbon through increased decomposition of root litter, particularly for sedge species with annual root turnover (Sullivan et al. 2007). Long‐term vegetation changes in response to a warming climate could also be influenced by competitive advantages below ground; for example, species able to forage deeper and for longer in permafrost soils could benefit as permafrost soils thaw (Hewitt et al. 2019; Pedersen et al. 2020), while the expansion of some species could be promoted by the warming‐enhanced development of ectomycorrhizal networks (Deslippe et al. 2011). Quantifying rooting phenology strategies across microclimates and plant communities will allow us to better predict future changes in below‐ground growth patterns and corresponding changes in carbon and nutrient cycling dynamics in warming tundra ecosystems (Smithwick et al. 2014; Warren et al. 2015).

Above‐ground productivity and phenology are influenced by both macro‐ and micro‐environmental variables, including snowmelt timing and soil, surface, and air temperatures (Assmann et al. 2019; Høye et al. 2007; Panchen and Gorelick 2015; Wookey et al. 1993), yet these same drivers could have less influence below ground (Abramoff and Finzi 2016; Liu et al. 2021). Experimental warming studies at tundra sites have indicated that the duration of below‐ground growing seasons for some species is largely unresponsive to factors that influence above‐ground phenology, such as snowmelt timing or warming (Möhl et al. 2022). However, while the overall length of the below‐ground growing season might not change, the timing of peak fine‐root growth could be shifted, for example, to later in deeper and warmer soils as permafrost thaws (Blume‐Werry et al. 2019). Root phenology could be influenced to some degree by late‐season timings of permafrost thaw, in particular for those species able to forage deeper to access the active layer thaw front (Blume‐Werry et al. 2019; Hewitt et al. 2019; Salmon et al. 2018). Variations in temperature across heterogeneous landscapes in a space‐for‐time setup could inform our understanding of change over time with warming (Ma et al. 2022; Radville et al. 2018; Schwieger et al. 2018).

Abiotic and biotic (e.g., nutrient hormone allocation) controls could differ between above‐ and below‐ground plant tissue (Abramoff and Finzi 2015; Liu et al. 2021; Ma et al. 2022). However, we lack paired above‐ and below‐ground phenology observations across communities and local temperature variation to test the extent of decoupling between drivers. Here, we combined leaf phenology observations from time‐lapse camera imagery with fine‐root growth metrics collected from across five tundra sites and 39 individual plots to compare the relative timings of plant tissue growth and senescence both above‐ and below‐ground. We used an in‐growth core field experiment to analyze root growth patterns across local temperature gradients to determine how root growth varies across warmer versus colder below‐ground conditions across the growing season. We investigated root growth dynamics across graminoid‐ versus shrub‐dominated plant communities to quantify different root phenological strategies among vegetation community types that are increasing in abundance in tundra ecosystems. Analyzing differences in leaf‐ and root phenology across microclimates provides a useful space‐for‐time comparison whereby warmer areas, in comparison to cooler areas, act as a natural proxy for future climate warming. Analyzing root growth patterns among community types will inform how tundra vegetation change could influence below‐ground fine‐root productivity, and ultimately carbon cycling (Bjorkman et al. 2020; Heijmans et al. 2022; Myers‐Smith et al. 2011; Niittynen et al. 2020).

In this study, we address the following research questions:

**RQ1: Is there above‐ versus below‐ground asynchrony in phenology, and if so, how does it vary across microclimates and community types?** Site‐specific studies indicate that the below‐ground growth of tundra plants extends beyond the period of growth above ground (Blume‐Werry 2021; Blume‐Werry et al. 2016; Radville et al. 2016). Therefore, we predicted that root growth would continue as the leaf tissue above ground was senescing and that this asynchrony would be greater in warmer microclimates versus colder microclimates. At sites with permafrost, if deeper active layers increased the overall volume of available soil in which roots could grow throughout the growing season, root growth could be greater in warmer microclimates. There could be a lag between above‐ground phenology and below‐ground phenology because soil temperatures lag behind air temperatures and thaw progressively across the summer, which could influence the timing of root production and foraging. If asynchrony is detected but is not explained by local temperature variation, plant community type could be the primary driver, particularly if there is clear differentiation in rooting strategy among plant functional types.

**RQ2: Is root productivity higher and the period of root growth longer in warmer versus cooler parts of the landscape?** Microclimates influence the growth of tundra plants, with greater productivity in warmer versus colder microclimates (Blume‐Werry 2021; Liu et al. 2021). We predicted that there would be higher fine‐root production in the warmer versus cooler parts of the landscape, leading to higher newly produced root biomass in the warmer plots within each site (Sullivan et al. 2007). We expected that root growth would extend in the warmer versus cooler plots within each site.

**RQ3: How does plant community type control below‐ground plant biomass and phenology?** Different plant functional types have different root growth strategies and can differentiate the timing of root foraging to acquire water and nutrients from permafrost soils (de Kroon et al. 2012; Pedersen et al. 2020). We predicted that graminoid‐dominated communities could exhibit root growth later in the season than shrub‐dominated communities as they are deeper‐rooting and could access nutrients released later in the summer by thawing permafrost and/or from deeper soil layers as they thaw or after shallow soil layers become nutrient depleted.

## 2 Materials and Methods

### 2.1 Site Selection

**FIGURE 1:**
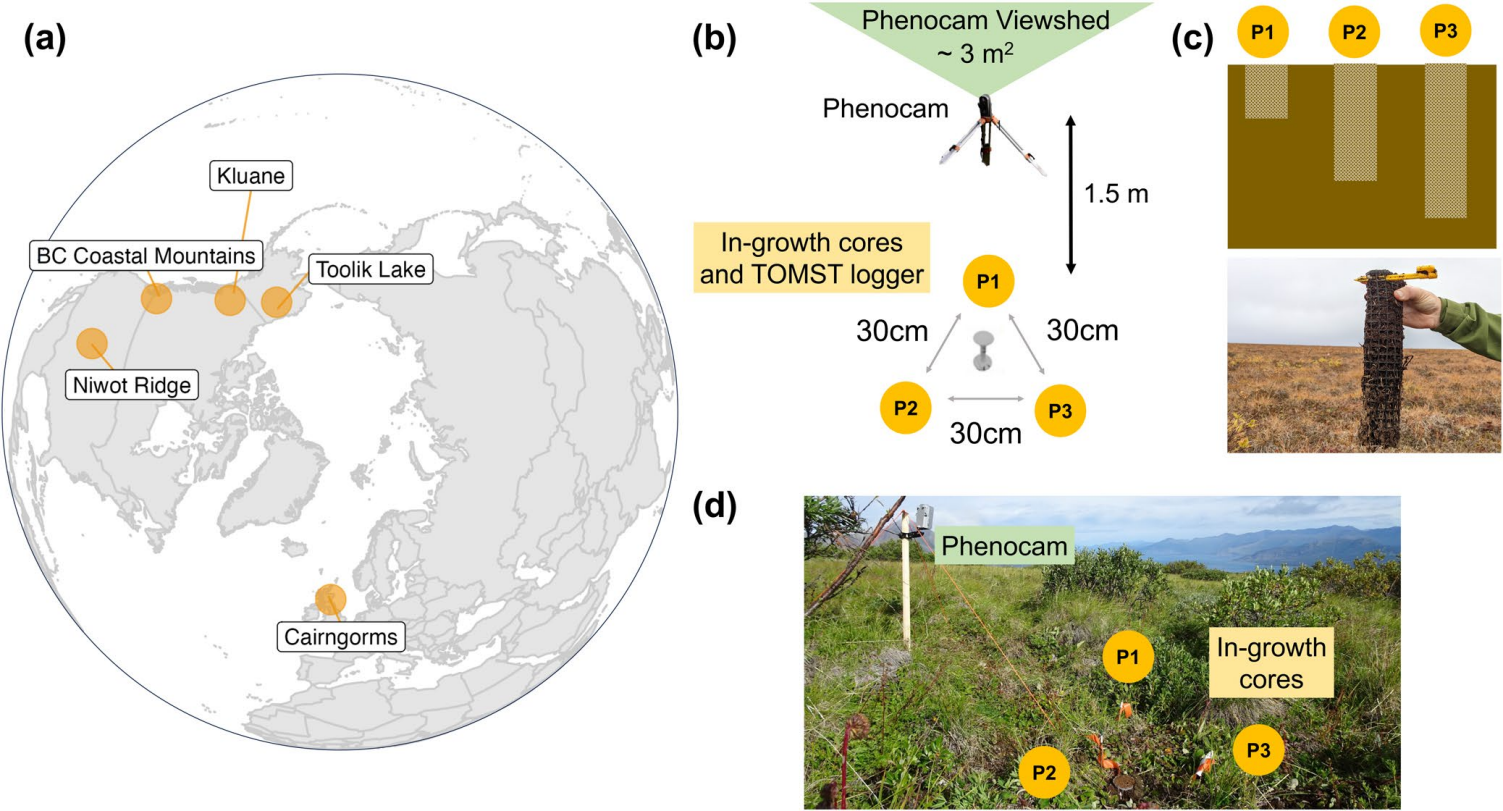
Our study included five sites, each with between 5 and 12 plots which contained paired phenocams and in‐growth cores. (a) Polar projection map of the five Arctic, subarctic and alpine tundra sites included in this study. Map lines delineate study areas and do not necessarily depict accepted national boundaries. (b) Birds‐eye‐view schematic of the subplots, showing the location of in‐growth cores P1, P2 and P3 in relation to the phenocam and the TOMST microclimate logger. (c) Cross‐section schematic of the differential in‐growth core depths in the soil profile at sites with permafrost (sites without a shallow thaw depth in the first half of the season and permafrost had the same depth for all cores). Photograph of a P3 core removed from Toolik in 2022 (Image Credit: Ruby An). (d) Photograph of Kluane Subplot 8 with a phenocam pointed northwards, alongside three buried in‐growth cores in summer 2021 (Image Credit: Madelaine Anderson).

We studied five tundra biome sites including Arctic tundra (Toolik, Alaska, USA), Subarctic alpine tundra (Kluane Lake, Yukon, Canada), and alpine/subalpine meadow (bc Coastal Mountains, bc, Canada; Niwot Ridge, Colorado, USA; Cairngorms Mountains, Scotland, UK). These sites spanned a wide geographical and climatological range (Figure 1a; Table S1). Each site also spanned a range of microenvironmental gradients and included a combination of graminoid‐dominated, shrub‐dominated, and mixed‐species communities, which we classified using site‐specific metadata, in situ observations, and phenocam observations (Table S1). Each site was outfitted with in‐growth cores, phenocams, and temperature monitoring. Temperature monitoring was generally with either a paired TMS‐4 TOMST, HOBO MX2201 Pendant, or Decagon RT1 thermistor environmental logger; however, in some plots nearby microclimate loggers were used to represent more than one plot.

Across sites, the phenocam and in‐growth core plots were located according to a selective gradient approach to ensure coverage of sites across different microclimates, graminoid‐dominated, shrub‐dominated and mixed‐species plant community types, and elevations. In the Cairngorms, the plots were established above the 500 m treeline along an elevational gradient on the west‐facing slope of the Allt a' Mharcaidh catchment. At Toolik Lake, Kluane, Niwot Ridge and the british columbia Coastal mountain sites, the in‐growth core plots were established alongside phenocams previously set up by collaborators for other research projects. We acknowledge that installing the experiment in areas with existing phenocam equipment could have introduced some additional variability among study areas.

### 2.2 In‐Growth Core Construction

We elected to use an in‐growth soil core approach rather than minirhizotrons (the observation tool most commonly used in below‐ground phenology studies in non‐Arctic environments), because minirhizotrons do not work in ice‐rich soils with substantial frost heave and freeze–thaw dynamics (Iversen et al. 2012). We constructed in‐growth peat cores with a diameter of 7‐cm using plastic meshing (rigid garden netting or industrial mesh tubing) with mesh holes no wider than 1 × 1 cm. Each core was filled with sterilised milled peat from garden centres local to the study sites (Table S1). We packed the milled peat into the in‐growth cores tightly to achieve similar densities among cores. At sites with permafrost (Table S1), in each cluster of three cores (hereafter, *plot*), the cores were divided into lengths of 10 cm (Phenology 1, or ‘P1’), 20 cm (Phenology 2, or ‘P2’), and 30 cm (Phenology 3, or ‘P3’). These different core lengths accounted for the differing active layer depths across the growing season in the summer of core removal such that the P1 cores could be removed early in the growing season when the active layer of thawed soil was shallow. At sites without permafrost, all cores had the same depth based on the soil depth at each site (between 15 and 20 cm). We recorded the weight and length of the cores at each site prior to deployment in the field.

### 2.3 Core Installation

At each site in the summers of 2021 and 2022, we separated the cores into plots (one plot = one × P1, one × P2, one × P3) and chose site locations whereby a minimum of five plots (15 cores in total) were distributed along environmental gradients specific to those sites, including soil moisture gradients, shrub versus graminoid‐dominated communities, and elevational gradients (Table S1). We recorded the geographic location of each site/plot using GPS devices available to contributors across sites. The core installation process took place at the end of the above‐ground growing season at all sites to ensure limited root growth in the year of installation as well as the deepest possible active layer thickness in sites underlain by permafrost (see Table S1 for 2021 core installation dates). At all sites other than Niwot, cores were installed 1.5 m behind each phenocam (see Figure 1b), instead of within the phenocam viewsheds, to mitigate destruction of the plant communities observed within the phenocam plots. At Niwot, cores were installed within 1.5 m of the phenocam within the viewshed to avoid disrupting existing temperature sensors behind the phenocams.

At each plot, the three cores were buried 30 cm away from one another in a triangular arrangement (see Figure 1b). Using a soil auger, we took a core of up to 30 cm depth (depending on the phenology removal grouping of the core; i.e., 10 cm for P1, 20 cm for P2 and 30 cm for P3) and recorded from this core the depth (cm) from the top of the core from at which the organic material transitions to a sandy or silty layer, a qualitative description of the soil type and density (e.g., ‘loose loamy’ or ‘dense clay’), and the depth (cm) from the top of the core of maximum rooting. We gently placed the peat‐filled in‐growth cores into the boreholes, making sure the base of the core reached the bottom of the hole and that there was no mesh extending upwards from the surface of the hole.

At each plot, we labelled the cores with a unique ID on a small flag or stake. In the centre of each plot, we installed microclimate loggers that logged temperature at −8, +2, and +15 cm from the surface (TMS‐4), 0 cm from the surface (HOBO MX2201 Pendant and Decagon RT1 thermistors) over the course of the experiment. The TMS‐4 loggers recorded temperatures at 15‐min intervals, while the HOBO and Decagon RT1 loggers recorded temperatures at 10‐min intervals. We aggregated this data into daily means spanning the period from 1 June 2022 to 31 August 2022. For each of the sites, we used the daily microclimate logger data to calculate June–August 2022 surface temperature data means, which we then categorised into quantile groupings to generate comparable groupings of the relative coldest (quantile 1), cool (quantile 2), warm (quantile 3), and warmest (quantile 4) areas across the landscape at each site. We intended initially to use soil temperature (−8 cm) data to better represent below‐ground climate conditions. However, the soil temperature readings were corrupted due to intermittent logger failures at some plots in two (Toolik, Niwot Ridge) of the five sites, so we used July and August surface temperature (+2 cm) for consistency across sites and microclimate datasets. These intermittent logger failures were not the result of displacements of the loggers from soil due to freeze– thaw dynamics or wildlife and did not affect the +2 cm and + 15 cm sensors within the loggers.

### 2.4 Phenocam Installation

At all sites apart from Niwot (Figure 1d; Table S1), we installed time‐lapse cameras (Moultrie Wingscape TimelapseCam Pro; or ‘phenocams’) at the location of each plot where possible. We affixed the phenocams to sturdy metal tripods at a height of 1 m above the ground. The phenocams pointed northwards to avoid direct sunlight and prevent glare, allowing the cameras to capture snow melt timing and the landscape greenness over the course of the growing season. We set the cameras to infinite focus and set them to capture one photograph per hour or four photographs per day at the highest pixel resolution possible for each camera. We installed these phenocams in 2021 when burying the cores, programmed them to collect imagery over the winter and following summer, and downloaded the data at the end of the growing season once the last core (P3) had been removed from each plot. The pre‐existing cameras at Niwot were programmed to take photos once every 30 min and were affixed to posts of about 2 m due to higher snowpack at the site.

### 2.5 Core Removal

The summer following core installation (i.e., 2022 when cores were installed in 2021), we removed the cores from the plots at staged intervals. We collected the P1 cores at the beginning of the growing season (shortly after snowmelt), the P2 cores at the middle of the growing season (corresponding with peak above‐ground productivity), and the P3 cores at the end of the growing season (before the return of snow). Due to logistical constraints and site‐specific productivity differences, the removal dates varied across sites but were consistent within sites. In addition, the temperature logger data and phenocam images were downloaded at the end of the growing season. Upon removal, the cores were immediately frozen to prevent root decomposition, and at the end of the growing season, all cores were shipped to the University of Edinburgh for laboratory analysis.

### 2.6 Laboratory Analysis

After thawing each of the frozen cores for 24 h in a refrigerator, we sub‐sectioned each core into distinct depth increments from surface to base (0–5 cm, 5–15 cm, 15–25 cm and 25–30 cm as appropriate for overall length). We recorded the full weight of each core and the full weight of each of these subsections. In addition, we recorded the weight of a wet soil subsample from the 0–5 cm increment of each core before drying them in an oven at 60°C for 72 h, and then recorded the weight of the dried subsamples. We used the ratio of these two weights to calculate the bulk densities of each of the depth increments, whereby:

(1a) $$\begin{array}{c} \begin{array}{c} {\text{BD}}_{\text{wet}}=W/V\\ {\text{BD}}_{\text{wet}}=\mathrm{wet}\;\text{weight bulk density}\\ \begin{array}{c} W=\mathrm{wet}\;\text{weight of ingrowth core depth increment}\\ V=\text{cylindrical volume of ingrowth core depth increment} \end{array} \end{array} \end{array}$$

(1b) $$\begin{array}{c} {\text{BD}}_{\text{dry}}={\text{BD}}_{\text{wet}}\times \left({\text{W}}_{\text{ds}}/{\text{W}}_{\text{ws}}\right)\\ {\text{BD}}_{\text{dry}}=\mathrm{dry}\;\text{weight bulk density}\\ \begin{array}{c} {\text{W}}_{\text{ds}}=\mathrm{dry}\;\text{weight of soil subsample}\\ {\text{W}}_{\text{ws}}=\mathrm{wet}\;\text{weight of soil subsample} \end{array} \end{array}$$

For each depth increment, we used tweezers to extract all of the roots less than 2 mm in diameter (i.e., the ‘fine roots’ that are most similar to leaves in their function of resource acquisition) within the soil and used distilled water to clean off the excess peat. We separated the roots into petri dishes based on morphological and colour differences. Once cleaned and separated by group and depth increment, we scanned each of the root groups using an Epson Perfection V850 scanner with an inbuilt wet tray, in 16‐bit grayscale and using an 800 dpi resolution. After scanning each root type by depth increment, we then placed the roots in metal tins and dried them in an oven at 60°C for 72 h, and then recorded the weight using a fine scale.

We summed the overall newly produced root biomass for each depth increment, before calculating root biomass density (i.e., root biomass per unit soil volume g cm^−3^). We calculated a daily root growth rate over the course of the growing season for each plot using the following equation:

(2) $$R=\frac{P3_{\text{rd}}-P1_{\mathrm{rd}}}{P3_{\text{doy}}-P1_{\text{doy}}}$$

R=Root biomass growth rate, 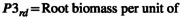 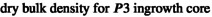, 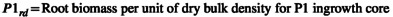, 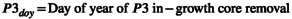, 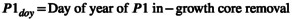.

Cores varied in length across sites due to site‐specific differences (i.e., soil quality, depth, presence or absence of permafrost) and in the timing of extraction (due to the timing of site‐specific permafrost thaw, snow melt, and snow return). To examine the differences between whole‐core root biomass versus distinct sections of the soil depth profile, we plotted mean root density for the full cores to compare against the mean root density from only the top 5 cm of the cores (Figure S2) and ran alternate versions of the statistical analysis using data from just the top 0–5 depth increments of each of the cores (Table S3). In this article, we present both sets of results but focus on the whole‐core data because these data better capture the full rooting depth of each sample (see protocol: Freschet et al. 2021).

### 2.7 Phenocam Analysis

We manually browsed phenocam images sequentially for each plot and recorded the day of year for the first occurrence of the following phenophases: plants first visible through snow, 90% snow melted, first 100% snow‐free day, first green leaf, 50% leaves green, 100% leaves green, first senesced leaf, 50% leaves senesced, 100% leaves senesced, first end‐season snow return, 50% end‐season snow cover, 100% end‐season snow cover. These thresholds were all visually assessed, a method which has been found to reliably replicate in situ field observations (de Fälthammar Jong (n.d.); Richardson 2023). We made these observations at the community level (i.e., across the entire viewshed of the phenocam) to ensure consistency across all sites and to generate proxies of greenness that we could use to interpret above‐ground productivity and the timing of both green‐up and senescence.

We used a combination of phenocam imagery, metadata from collaborators, and scanned root images to qualitatively classify the plots into graminoid‐dominated, shrub‐dominated, or mixed‐species community groupings. Finally, we calculated a “synchrony metric” for each core plot to estimate the percentage of total root growth that had occurred per plot between the first in‐growth core removal date (P1) and the date of peak above‐ground growth for each plot, relative to the maximum root growth from stage P3. The metrics were then zero‐centered to compare across sites. This metric represents a coarse estimate of root growth accumulation by the time of peak above‐ground greenness relative to the total root accumulation observed in the P3 cores (see Figure S1). Therefore, the metric is more comparable within sites (i.e., all of the P1 and P3 removal dates are consistent at each location), but is not as comparable among sites (i.e., P1 and P3 removal dates varied between, for example, Toolik and Niwot Ridge) and cannot be considered a full assessment of above‐ and below‐ground growth asynchrony.
(3)

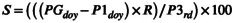


S=Synchrony Metric=%Root Growthatdate of100%Greening, 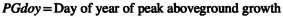 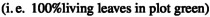, 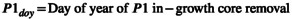, 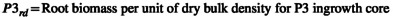, $R=\text{Root biomass growth rate}\;\left(\text{accounting for}\;P1\;\text{to}\;P3\;\text{growth rate}\right)$.

We also calculated specific P1‐P2 and P2‐P3 root growth rates to distinguish any accelerations between time periods. However, due to the differential timing of P2 removals among sites (i.e., the removals were not always exactly at the time of the above‐ground mid‐season), we chose not to include these in any statistical analyses, but have instead visualised the results in Figure S4.

### 2.8 Statistical Analysis

We used Bayesian linear models to run three sets of regression analyses: (1) one set examining the variation of newly produced root biomass across microclimates and plant communities, (2) one set examining the variation in root growth rates across microclimates and plant communities, and (3) one set examining the variation of our derived synchrony metric across microclimates and plant communities.

We used the ‘*brms’* package (Bürkner 2017) in *R* version 3.6.6 (R Core Team 2013) and fitted each of the models with weakly informative priors (half Student‐t priors with three degrees of freedom), with three chains of 4000 iterations each and a warmup of 1000 iterations. To assess model convergence, we examined Bayesian trace plots and posterior predictive fits, and checked to ensure that R_hat_ values (ratio of effective sample size to overall number of iterations) were all close to 1.00.

For the right‐skewed root biomass data, we set the distribution family to ‘skew_normal’ in brms. For each model, we included ‘community type’ and ‘microclimate quantile’ as ordered categorical fixed effects, and for the biomass model alone we included the removal stage (P1, P2, P3) as a categorical fixed effect to examine the differences in root biomass development across in‐growth core removal intervals. For the first set of models (examining how biomass varied across microclimates and plant community types) we included an interaction term between the removal stage and plant community type, to quantify whether different plant communities produced roots at different harvesting stages during the growing season. Microclimate and community type do not co‐vary strongly at these sites (Figure S3).

To account for differences in environmental characteristics and in‐growth core materials used among sites, we included “site” as a random intercept term in our statistical models. We intended to include random slopes in the model design to allow for different relationships between root phenology variables and the fixed effects, but ultimately removed this model structure due to a lack of model convergence. To test whether similar results emerged using continuous microclimate data instead of quantiles, we ran an additional set of models with the same parameters but with continuous daily June–August surface temperatures, zero‐centered within each site, in place of the climate quantile metric. All code and data used in this analysis is available to review and download (Gallois 2025).

## 3 Results

**FIGURE 2:**
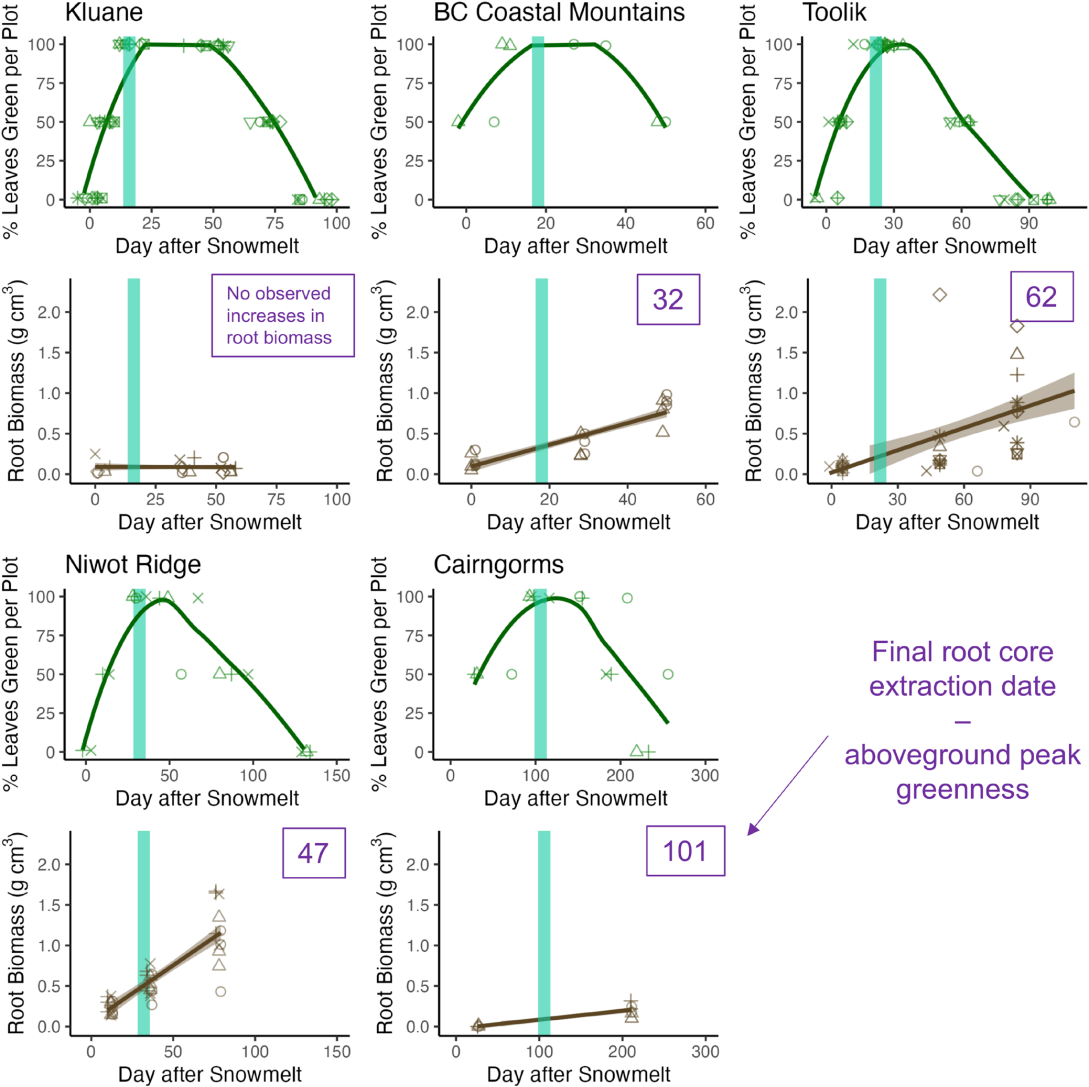
Root growth continued after above‐ground plant tissues began to senesce across all but one site. For each site, the top panel represents phenocam‐derived greening curves, with each green point representing the date after 100% snowmelt per plot that a recorded phenophase occurred (bud burst, 50% green leaves, 100% green leaves, first yellow leaf, 50% yellow leaves, and 100% yellow leaves). Green trend lines were generated using the loess smoothing feature in ggplot2. For each site, brown points in the bottom panel represent the root biomass per g cm^−3^ of soil volume averaged across each in‐growth core corresponding to their extraction from the experiment and the timing of that extraction in relation to the date of 100% snowmelt per plot. For each site, both green and brown points were assigned shapes to represent the corresponding phenocam for each soil core. Brown trend lines were generated using linear regression. Blue‐green vertical lines represent the site‐averaged dates of peak above‐ground growth, or the mean ‘day after snowmelt’ that plots reached 100% green leaves. Sites are ordered here by time taken to achieve full green‐up, from fastest (Kluane) to slowest (Cairngorms). Purple numeric labels on the bottom panel indicate the number of days of observed root growth beyond the date of peak above‐ground productivity (date of P3 extraction minus the date of peak aboveground greenness), excluded for Kluane because there was no observed root biomass increase over time at this site. See Table S1 for 2022 core removal dates.

We found that root growth continued for at least 56 days (on average) after the date of peak above‐ground productivity at each site (Figure 2). Our estimate of the timing of root growth likely underestimates the full below‐ground growing season, as we did not collect any additional below‐ground data before the start, and beyond the end of our respective field expeditions. Calculated as the period of time relative to the first date of above‐ground leaf yellowing, newly produced root biomass continued to increase for at least 62 days (or 74%) after the onset of above‐ground senescence at Toolik, 32 days (64%) in the bc coastal mountains, 60 days (47%) at Niwot Ridge, and 101 days (48%) in the Cairngorms. Meanwhile, there was no detectable increase in root biomass over time at Kluane, potentially due to the scarcity of core extractions during the above‐ground senescence period (Figure 2). Across sites, we did not find any difference between above‐ and below‐ground synchrony with local temperature variation or among plant communities (Table S2). While there were no significant differences in synchrony between graminoid‐dominated and shrub‐dominated communities, we found that the proportion of total newly produced root biomass at the time of peak above‐ground greenness was considerably higher for graminoid relative to mixed‐species communities (−7.59 g cm^3^, CI: −11.22 to −3.87).

**FIGURE 3:**
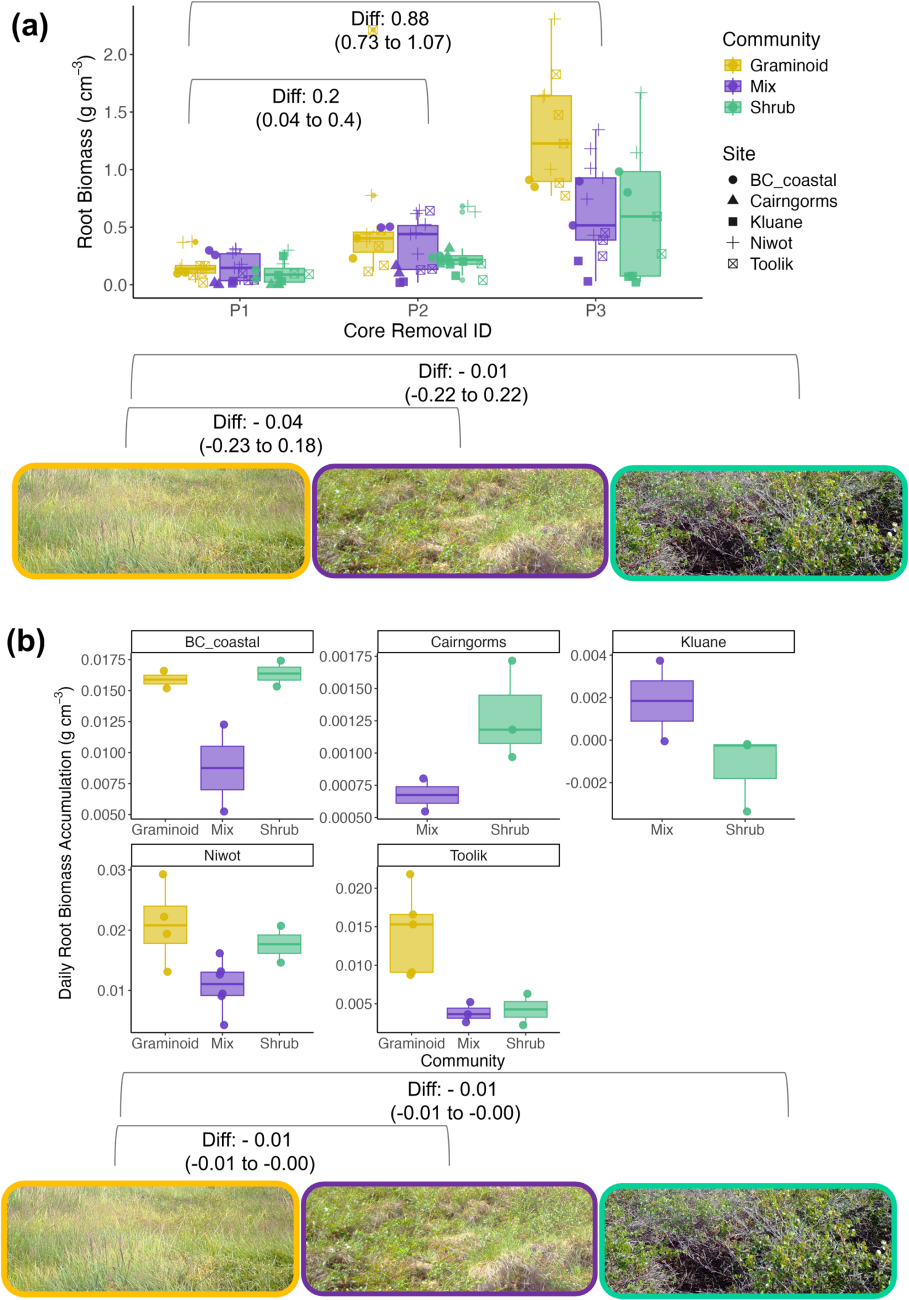
(a) Root biomass accumulation was greater for graminoid‐dominated relative to shrub‐dominated and mixed‐species plots. Error bars represent the distributions of the root biomass per soil volume (g cm^−3^) for each stage of removal (P1, P2 or P3) across the three community types: Graminoid‐dominated, mixture of graminoid and shrub, and shrub‐dominated. Points represent the root biomass per g cm^−3^ of soil volume averaged across each in‐growth core Annotations in the box plot denote the difference estimates of root biomass between the removal stages (g cm^−3^) with 95% credible intervals provided in parentheses. Annotations on the photography panel denote the difference estimates of root biomass among the vegetation community groups (g cm^−3^) with 95% credible intervals provided in parentheses. (b) Root growth rates were generally faster in the graminoid‐dominated plots than the shrub‐dominated or mixed‐species plots. Error bars represent the distributions of the daily root biomass accumulation (g cm^−3^) across the summer among the three community types. Points represent the daily root biomass accumulation per g cm^−3^ of soil volume averaged across each in‐growth core plot. Annotations on the photography panel denote the difference estimates of root growth rate among the vegetation community groups (g cm^−3^) with 95% credible intervals provided in parentheses. Photos are select screenshots from 9 July 2021 across three Toolik plots representing the corresponding community types (Image Credits: Ruby An). See Table S2 for full statistical output.

Newly‐produced root biomass varied significantly by community type across the sites at the final (P3) harvest (Figure 3a; Figure S2a; Table S2). We found that between the P1 and P3 harvesting intervals, in‐growth cores from graminoid‐dominated communities had 129% higher root biomass than shrub‐dominated communities (categorical difference of 0. 55 g cm^−3^, CI: 0.29 to 0.79) and 130% higher biomass than mixed‐species communities (categorical difference of 0. 53 g cm^−3^, CI: 0.27 to 0.83). In comparison, the differences in root biomass between the P1 and P2 harvesting intervals were minimal between plant community types. Likewise, daily root growth rates (i.e., rate of daily root growth as calculated between first and last core harvest; Table S2b; Equation 2) were faster in graminoid, relative to mixed and shrub‐dominated plant communities (Figure 3b; Figure S4; Table S2), with in‐growth cores installed in graminoid‐dominated plots exhibiting daily root growth rates 84% faster than shrub‐dominated communities (shrub slope: −0.01 g cm^−3^ per day, CI: −0.01 to −0. 006), and 42% faster than mixed‐species communities (mixed slope: −0.01 g cm^−3^ per day, CI: −0.01 to −0. 003).

**FIGURE 4:**
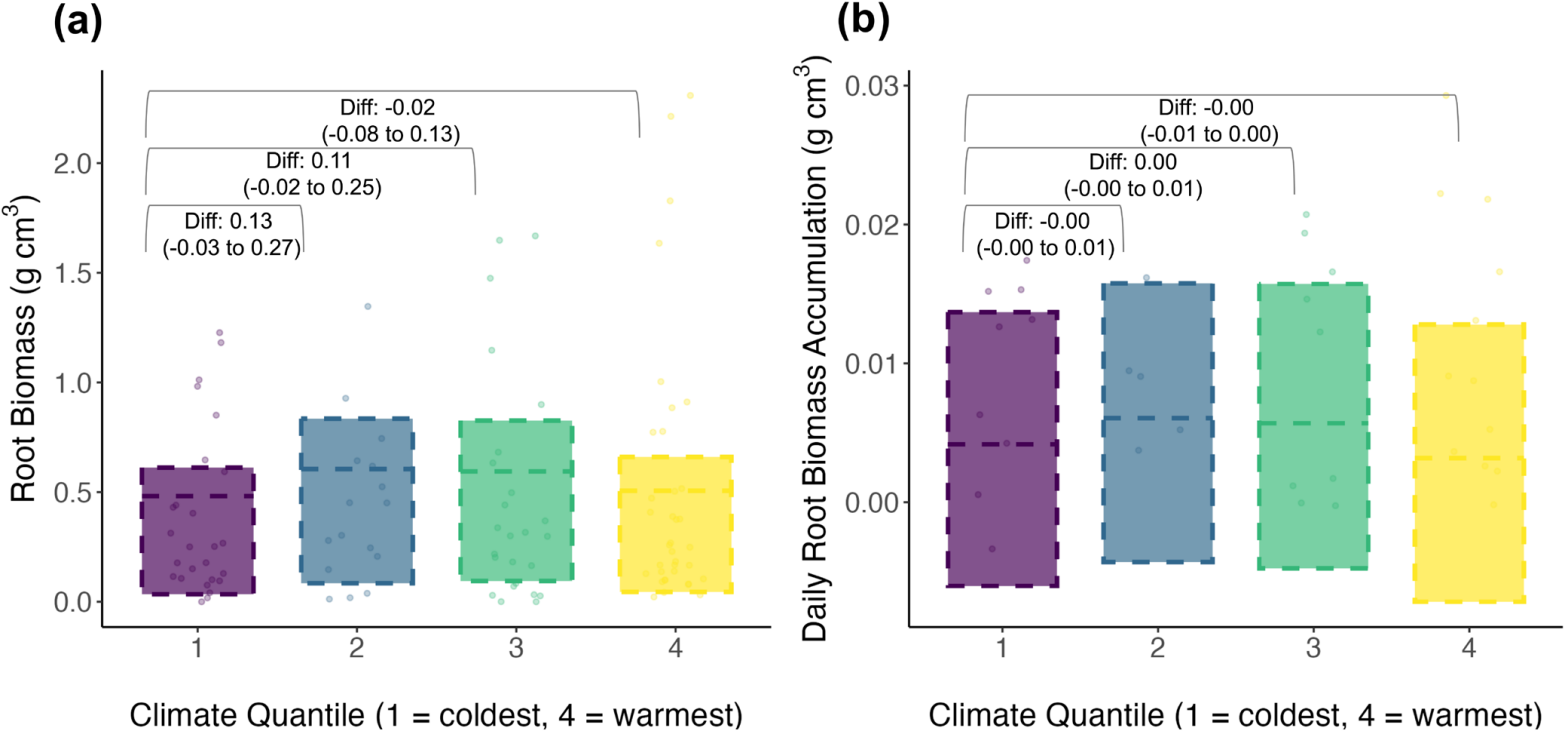
Root biomass allocation and root growth rates did not correspond with local soil surface temperatures. Error bars in (a) represent the modelled distributions (Table S2a) of the root biomass/soil volume (g cm^−3^) for the final stage of removal (P3), plotted across summer surface temperature microclimate quantile groups. Error bars in (b) represent the modelled distributions (Table S2b) of the daily root growth rates between P3 and P1, plotted across summer surface temperature microclimate quantile groups. Points represent the root biomass per g cm^−3^ of soil volume averaged across each in‐growth core. Annotations denote the difference estimates of root biomass (a) and root growth rate (b) (g cm^−3^) with 95% credible intervals provided in parentheses. See Tables S2 and S3 for full statistical output.

Contrary to our predictions, newly produced root biomass did not vary across microclimates (Figure 4; Table S2a). The difference in root biomass per soil volume between the coldest and warmest microclimate groupings was −0.023 g cm^−3^ (−0.084 to 0.134). Similarly, daily root growth rates (i.e., the daily rate of root growth as calculated between the first and last core harvest) across the growing season were not significantly different across surface temperature quantiles (Table S2b). For example, the difference in root growth rate per day between the coldest and warmest quantile groupings was −0.0001 g cm^−3^ day^−1^ (−0.006 to 0.003). For all model designs, the top 5‐cm only model results revealed the same trends. Likewise, for all model designs, there was a consistent lack of correspondence between continuous daily surface temperature observations and all root growth metrics (Tables S3–S5).

## 4 Discussion

Above‐ground leaf phenology and below‐ground root phenology were asynchronous across all sites except Kluane, with root growth continuing long after above‐ground peak productivity as assessed by peak leaf greenness (Figure 2). At some sites, there was evidence that the below‐ground growing season extended beyond the point of 50% above‐ground leaf senescence; although without continuous core removals later in the season, it was not possible to determine the time of root growth cessation (Figure 3). Our findings from five sites from the Western Arctic, North America, and Scottish alpine tundra correspond with studies from Arctic Sweden and Western Greenland (Blume‐Werry et al. 2016; Radville et al. 2018; Sullivan et al. 2007). We now have compelling evidence that above‐ and below‐ground tundra phenology is asynchronous and that the below‐ground growing season can extend 50% longer or more than the above‐ground growing season (Blume‐Werry et al. 2016; Radville et al. 2018; Sullivan et al. 2007). Importantly, vegetation community composition, rather than microclimate, had the greatest influence on the accumulation of newly produced root biomass and root growth rates. In particular, root biomass was greater and root growth rates faster in graminoid‐dominated relative to shrub‐dominated and mixed‐species plots (Figure 3). Additionally, we observed a distinct peak in root growth in graminoid‐dominated plots, usually taking place towards the end of the above‐ground growing season, while root biomass accumulated more linearly over time in the mixed‐species and shrub‐dominated plots (Figure 3; Figure S4). Contrary to our hypotheses, we found no correspondence between microclimate and root biomass accumulation, daily root growth rates, or above‐ versus below‐ground phenological asynchrony (Figure 4). This analysis therefore highlights that plant community types, rather than microclimates, could be the most important influence on root productivity and the timing of root growth in tundra ecosystems.

### 4.1 Root Biomass Was Higher—And Growth Rates Faster—In Graminoid Dominated Plots

Root biomass was greater, and daily root growth rates were faster in the graminoid‐dominated plots than in shrub‐dominated or mixed‐species plots (Figure 3; Table S3a). Many studies highlight different root growth strategies within and among plant functional types, often noting that graminoid species will forage later in the growing season and in deeper soils in order to access nutrients available at the permafrost thaw front (Keuper et al. 2017; McKane et al. 2002; Salmon et al. 2018; Sullivan et al. 2007). Annual root growth by sedge communities already contributes significantly to net primary productivity (NPP) in the tundra (Iversen et al. 2015; Sloan 2011; Sloan et al. 2013). In areas where conditions are projected to become more mesic and provide optimal habitat to support graminoid expansion (Andresen and Lougheed 2021; Heijmans et al. 2022), NPP could, therefore, increase. However, in areas where woody shrubs outcompete other plant species (Mekonnen et al. 2018), root biomass could be reduced, particularly at deeper soil depths close to the active layer thaw front. Different root biomass and growth characteristics are likely, therefore, to influence local and regional carbon flux dynamics in areas where tundra vegetation composition is predicted to reshuffle, potentially bringing carbon stores towards the surface with increasing shrub cover.

Daily root growth rates were significantly faster in graminoid‐dominated communities than in mixed‐species or shrub‐dominated communities (Figure 3; Table S2b), which was particularly defined by a visible graminoid growth peak towards the end of the growing season in comparison to a more linear growth rate in the other plots (Figure 3; Table S2b). This rapid increase in biomass in late summer could reflect enhanced uptake of nutrients by non‐mycorrhizal graminoid roots towards the end of the growing season when this abundant nutrient source is made available by thaw (Hewitt et al. 2019; Keuper et al. 2017; Pedersen et al. 2020; Wang et al. 2017). If this ability to harness nutrients late in the season is unique to deep‐rooting graminoid species, these results potentially challenge the assumption that shrubs have an exclusive competitive advantage in warming tundra landscapes (Mekonnen et al. 2018), emphasizing that rooting strategies differ greatly across plant communities. Niche differentiation in rooting depth and root phenology is driven in large part by differences in nutrient availability across the soil profile, with deeper‐rooting species able to access newly thawed nitrogen from the active layer in late summer, and shallower‐rooting species instead deriving nutrients from litter decomposition (or via symbiosis with mycorrhizal fungi) closer to the surface (McKane et al. 2002; Keuper et al. 2017). Shallow‐rooting shrubs and other snow‐bed species could also access nutrients from spring snowmelt, triggering early initial root growth and more gradual growth throughout the growing season (Wang et al. 2016; Onipchenko et al. 2014). While we did not explicitly examine nutrient content, it is likely that the plant community type and composition within our plots interacted strongly with different mechanisms of nutrient availability to drive root colonization depth and rates across the soil profile.

Associations between mycorrhizal fungi and roots can modulate nutrient acquisition in nutrient‐poor soils (Read 2003; Iversen et al. 2015). However, the mechanisms between these interactions and root phenology are poorly understood. Typically, mycorrhizal associations are stratified by depth (and by plant functional type) in tundra soils, with shrub roots more likely to have ericoid and ectomycorrhizal associations and graminoid roots more likely to be non‐mycorrhizal (Newsham et al. 2009; Iversen et al. 2015; Hewitt et al. 2019). Arbuscular mycorrhizal associations are found primarily in low Arctic and alpine tundra with grass, forb, and some shrub species, but not in sedges (Gardes and Dahlberg 1996). Deep‐rooting graminoids and forbs, particularly non‐mycorrhizal species (e.g., *Eriophorum vaginatum* ) more rapidly access nutrients released from thawing permafrost toward the end of the growing season, while shallow‐rooting mycorrhizal shrubs (e.g., *Betula nana* , *Salix* spp.) could more gradually take up nitrogen throughout the growing season with the symbiotic assistance of mycorrhizae (Hewitt et al. 2019). It is difficult to distinguish plant species by fine‐root morphology alone, and the lateral growth of tundra roots meant that we could not directly infer root provenance from the above‐ground vegetation adjacent to the in‐growth cores. As such, we cannot directly infer which of the species present in our plots had ectomycorrhizal or arbuscular mycorrhizal associations. However, the different root phenology dynamics highlighted in our study correspond closely to these species‐specific dynamics. It could be beneficial, therefore, to apply similar methods to research focusing on species‐specific mycorrhizal symbioses and their influence on below‐ground phenology.

### 4.2 Root Productivity and Phenology Did Not Correspond to Spatial Variation in Microclimate

Across these topographically heterogeneous tundra sites, root growth rates and newly produced root biomass did not vary consistently across surface temperature ranges within sites (Figure 4; Table S2). Previous research presents contrasting evidence on the influence of microclimate on root productivity and phenology in tundra ecosystems. For example, field studies using experimentally warmed plots often indicated that the timing of the start of the below‐ground growing season and the length of this growing season were generally unaffected by increased temperatures (Ma et al. 2022; Radville et al. 2018). Likewise, experimental snowmelt removal indicates that while advanced snowmelt often leads to an advanced above‐ground growing season, the timing of root phenology was largely unaltered (Blume‐Werry et al. 2017; Möhl et al. 2022). In contrast, Liu et al. (2021) found that the below‐ground growing season at a tundra site lengthened by approximately 2 days for each additional 1°C of warming. The timing of phenophases above‐ground appears to be driven jointly by variation in snowmelt timing and surface microclimatic conditions (Assmann et al. 2019; Jerome et al. 2021; Kelsey et al. 2021). Root phenology does not appear to have the same sensitivity to microclimate, which indicates the potential for further above‐ versus below‐ground asynchrony under climate warming scenarios.

Variation in permafrost conditions within and across sites could influence root growth dynamics and thresholds for soil temperature—phenology interactions. These five study sites varied in their permafrost status and depth to permafrost, with Toolik being underlain by ice‐rich permafrost, alpine sites being underlain by discontinuous mountain permafrost, and the more southerly Cairngorms site being underlain by bedrock. Root growth is often enhanced where thaw is deeper (Hewitt et al. 2019; Keuper et al. 2017; Pedersen et al. 2020). Active layer thickness in areas underlain by permafrost is highly spatially heterogeneous and typically deeper in correspondence with warmer air temperatures (Biskaborn et al. 2019; Yi et al. 2018). In alpine soils, root growth is strongly limited by soil temperature due to the cessation of cell elongation and differentiation below 0.8°C to 1.2°C (Nagelmüller et al. 2017; Sebastian et al. 2016). The mean summer soil temperature at 6 cm depth was over 5°C across all sites (Table S1, not including plots where logger readings were corrupted), so it is likely that the roots in this study were not subject to soil temperatures below their thermal tolerance in summer. It is also possible that above this thermal threshold of 0.8°C to 1.2°C, temperature no longer controls root growth patterns.

Tundra roots could be more strongly influenced by macro‐scale temperature variation than microclimate. The site with the warmest July–August surface temperatures (Toolik; Table S1) had the greatest end‐of‐season newly produced root biomass, while the site with the coldest summer surface temperatures (Kluane; Table S1) had the lowest end‐of‐season newly produced root biomass. For example, on decadal timescales, long‐term climate warming can promote increased total root biomass through increased litter decomposition, increased permafrost thaw, and increased nutrient mineralisation rates (Wang et al. 2017; Hill and Henry 2011; McKane et al. 2002; Keuper et al. 2017). While both the timing of core extractions and overall levels of biomass varied by site, it is possible that on a macro‐scale, if not a micro‐scale, warmer summer conditions could have prompted greater root growth at warmer sites.

### 4.3 Above‐ and Below‐Ground Phenology Are Not Synchronized in Tundra Communities

Above‐ and below‐ground root phenology was asynchronous across almost all sites, with root growth continuing up to 74% after the above‐ground peak in leaf phenology (Figure 3). However, we found no correspondence between microclimate and phenological synchrony (Table S2c). These findings directly support observations that the below‐ground growing season in tundra ecosystems can significantly extend beyond the above‐ground growing season, in accordance with studies in Arctic Sweden and Western Greenland (Blume‐Werry 2021; Blume‐Werry et al. 2016; Liu et al. 2021; Radville et al. 2018; Sullivan et al. 2007). Adding five additional sites to existing studies, our results provide a critical cross‐biome perspective. We have uncovered phenological asynchrony in Arctic and alpine tundra landscapes spanning a range of topographic and environmental gradients and highlighted that plant community type, more than microclimate, influences this asynchrony.

Plant phenology is intrinsically tied to carbon cycling in tundra ecosystems—with increased vegetation productivity increasing uptake of atmospheric carbon and longer growing seasons triggering increased respiration towards the end of the summer (Bruhwiler et al. 2021; Ueyama et al. 2013). The drivers of above‐ versus below‐ground phenology in the tundra could be decoupled, potentially as a function of internal nutrient and hormone allocation timings within plants (Abramoff and Finzi 2016) or via the varying physiological relevance of above‐ground conditions such as air temperature versus below‐ground conditions such as thaw depth for different tundra species (Liu et al. 2021). In areas where the above‐ground growing season advances and the below‐ground growing season extends long after peak leaf productivity, the total growing season incorporating both above‐ground and below‐ground plant components is therefore lengthened and elements of plant productivity functionally decoupled.

### 4.4 Scope for Future Research

While these results showcase clear asynchrony in root productivity and phenology among tundra vegetation community types, key questions remain. Firstly, we were only able to capture summer growing season dynamics during the snow‐free period in this study and could therefore not quantify root growth throughout the entirety of the potential below‐ground growing season, as we were not able to quantify the cessation of root growth. This means that we were not able to definitively quantify the true timing of the beginning and end of the below‐ground growing season. Furthermore, the ‘synchrony metric’ is therefore dependent on the timings of the P1 and P3 harvests, and is therefore more comparable within‐sites and less comparable across‐sites. However, there is evidence that root growth could be possible outside of the snow‐free period where photosynthesis and above‐ground growth are constrained by snow cover and light (Blume‐Werry et al. 2017; Riley et al. 2021). A priority for future research will be to investigate how much roots grow outside of the snow‐free season window, both before spring snowmelt and after autumn snow‐return as surface soils begin to freeze.

Our analyses revealed evidence of late‐season root‐growth ‘peaks’ in graminoid‐dominated plots, which could at some sites (such as Toolik) be exacerbated by permafrost thaw dynamics. Analysis of both thaw depth and root growth over the course of one growing season using finer temporal resolution could help identify whether graminoid root growth and rooting depth closely track the timing of active layer thaw (Blume‐Werry et al. 2019; Hewitt et al. 2019; Keuper et al. 2017; Shaver and Billings 1975), and pinpoint the extent to which these phenomena track above‐ground phenology. Future analysis could use the significantly varying below‐ground biomass and growth rate data alongside projections of future vegetation range shifts to scale up projections of both carbon uptake and carbon respiration from root systems in tundra ecosystems.

Differences in plant community type across a landscape within a relatively static timestamp are unlikely to equate to long‐term vegetation changes across decades. While it could be possible to infer below‐ground productivity and carbon cycling dynamics from above‐ground community observations, there could be confounding long‐term environmental interactions such as permafrost thaw, flooding and drought events, and changes to herbivore presence that are unaccounted for in this study. The methods we used for this study could be applied over multiple growing seasons to analyse the difference between above‐ and below‐ground phenology and root yield in warmer and colder years. Growth chamber experiments could additionally be used to gain a detailed understanding of how growth continues as soils freeze and thaw under warmer temperatures and lengthening growing seasons. Critically, extending these analyses across multiple years and a greater number of sites, and combining with other methods to capture below‐ground growth could further refine our understanding of how above‐ versus below‐ground growth asynchrony is changing spatiotemporally, and could allow us to more specifically identify the causal links between root phenology and both macro‐ and micro‐environmental conditions.

## 5 Conclusion

The tundra biome is undergoing a rapid shift in vegetation towards more shrub‐ and graminoid‐dominated plant communities as the climate warms (Berner and Goetz 2022; Bhatt et al. 2013; Elmendorf et al. 2012; Forbes et al. 2010; Myers‐Smith et al. 2011, 2020). Therefore, long‐term changes in vegetation community type could influence root biomass and root growth rates in the tundra with important implications for carbon cycling (Jones et al. 2009; Sokol and Bradford 2019). Our study has highlighted that root productivity varied significantly by plant community type, but not by microclimate. Furthermore, above‐ and below‐ground plant phenology was asynchronous across Arctic and alpine tundra sites, with root growth often continuing beyond the point of 50% above‐ground leaf senescence. The drivers of root growth and phenology remain critically understudied, and the importance of fine roots in tundra carbon cycling is commonly oversimplified in Earth system models (Blume‐Werry et al. 2023; Smithwick et al. 2014; Warren et al. 2015). The results from this study reveal a clear pathway toward modelling these changes by using above‐ground community composition to estimate below‐ground productivity and phenology.

## Author Contributions

**Elise C. Gallois:** conceptualization, data curation, formal analysis, funding acquisition, investigation, methodology, project administration, validation, visualization, writing – original draft, writing – review and editing. **Isla H. Myers‐Smith:** conceptualization, funding acquisition, investigation, methodology, project administration, resources, supervision, validation, writing – review and editing. **Colleen M. Iversen:** conceptualization, methodology, resources, writing – review and editing. **Verity G. Salmon:** conceptualization, methodology, resources, writing – review and editing. **Laura L. Turner:** conceptualization, data curation, funding acquisition, investigation, methodology, writing – review and editing. **Ruby An:** investigation, writing – review and editing. **Sarah C. Elmendorf:** funding acquisition, investigation, writing – review and editing. **Courtney G. Collins:** funding acquisition, investigation, writing – review and editing. **Madelaine J. R. Anderson:** investigation, writing – review and editing. **Amanda Young:** investigation, resources, writing – review and editing. **Lisa Pilkinton:** investigation, methodology, writing – review and editing. **Gesche Blume‐Werry:** investigation, writing – review and editing. **Maude Grenier:** conceptualization, investigation, methodology, writing – review and editing. **Geerte Fälthammar‐de Jong:** investigation, methodology, writing – review and editing. **Inge H. J. Althuizen:** investigation, writing – review and editing. **Casper T. Christiansen:** investigation, writing – review and editing. **Simone I. Lang:** investigation, writing – review and editing. **Cassandra Elphinstone:** investigation, resources. **Greg H. R. Henry:** investigation, resources. **Nicola Rammell:** investigation, resources. **Michelle C. Mack:** investigation. **Craig See:** investigation, writing – review and editing. **Christian Rixen:** investigation, writing – review and editing. **Robert D. Hollister:** investigation.

## Conflicts of Interest

The authors declare no conflicts of interest.

## Supporting information


Data S1.

## Data Availability

The data and code that support these findings are openly available in Zenodo at: Elise G. (2025). EliseGallois/Above_v_Below_Phenology: rootpheno_2025 [Data set]. Zenodo. https://doi.org/10.5281/zenodo.15024475 and GitHub at https://github.com/EliseGallois/Above_v_Below_Phenology.
