# Supplementary material for: Tundra Vegetation Community Type, Not Microclimate, Controls Asynchrony of Above‐ and Below‐Ground Phenology
Source: Glob Chang Biol. 2025 Apr 2;31(4):e70153. doi: 10.1111/gcb.70153 (PMC11964126; doi:10.1111/gcb.70153)
Supplement: Supplementary file 1 — Data S1. [file GCB-31-e70153-s001.docx]

# Supplementary Materials

**Table S1.** Site metadata summaries, including geographical location, soil type, site climate summaries, and vegetation properties.

| **Site Name** | **Coordinates (Lat, Lon)** | **Average July-Aug Surface Temperature (°C )** | **Elevation above sea level (metres)** |  | **2021 Core Installation dates**  **(Day of Year)** | **2022 Core removal dates (Day of Year)** | **# Plots (Clusters containing 3 x soil cores)** | **Subplot: Phenocam pairings** | **Vegetation Properties** | **Milled Peat Type** | **Notes on microclimate logger sharing** |
| --- | --- | --- | --- | --- | --- | --- | --- | --- | --- | --- | --- |
| **Kluane Plateau** | 61.28, -138.93 | 6.8 | 1200-1400 |  | 225 | P1: 173 P2: 208, P3: 225 | 5 | 4:KP4, 5:KP5, 7:KP7, 8:KP8, 9: KP9 | Shrub dominated and mixed-species plots | Golf Green Sphagnum Peat Moss | No shared loggers |
| **Toolik Lake** | 68.63, -149.59 | 14.3 | 730 |  | 230 | P1: 167, P2: 211, P3: 246 | 10 | TFS1: TL_01, TFS2: TL_02, TFS3: TL_03,  TFS4: TL_04,  TFS5: TL_05,  TFS6: TL_06,  TFS7: TL_07,  TFS8: TL_08,  TFS9: TL_09,  TFS10: TS_11 | Graminoid dominated, shrub dominated, and mixed-species plots | Sunshine Canadian Peat Moss | Logger 94213648 was shared by plots TFS7-10. |
| **Niwot Ridge** | 40N, -105W | 10.8 | 3050 |  | 237 | P1: 171, P2: 195, P3: 237 | 12 | 11 a, b and c plots: sn_11, 12 a, b and c plots: sn_12, 13 a, b and c plots: sn_13, 8 a, b and c plots: sn_09 | Graminoid dominated, shrub dominated, and mixed-species plots | Golf Green Sphagnum Peat Moss | Logger sn_08 was shared by plots 8A, 8B, and 8C**;**  sn_11 was shared by plots 11A, 11B, and 11C**;** sn_12 was shared by plots 12A, 12B, and 12C**;** sn_13 was shared by plots 13A, 13B, and 13C. |
| **BC Coastal Mountains** | 50.04, -123.19 | 7.1 | 1430 - 1450 |  | 226 | P1: 211, P2: 239, P3: 260 | 6 | All Brandywine plots: BRA_BC, All Tricouni plots: TRI_BC | Graminoid dominated, shrub dominated, and mixed-species plots | Golf Green Sphagnum Peat Moss + Promix Peat Moss (mixed) | Logger **94221281** was shared by plots **Tricouni_1** and **Tricouni_2.** |
| **Cairngorms** | 57.07, -3.49 | 13.6 | 325 - 1111 |  | 310 | P1: 127, P2: 311 | 6 | INT Plots: CRN_SN, SUM Plots: CRN_Snowcam, TR Plots: CRN_TL | Shrub dominated and mixed-species plots | Jamieson Brothers Irish Peat Moss | Logger **20907955** was shared by plots **TR2** and **TR1.** |


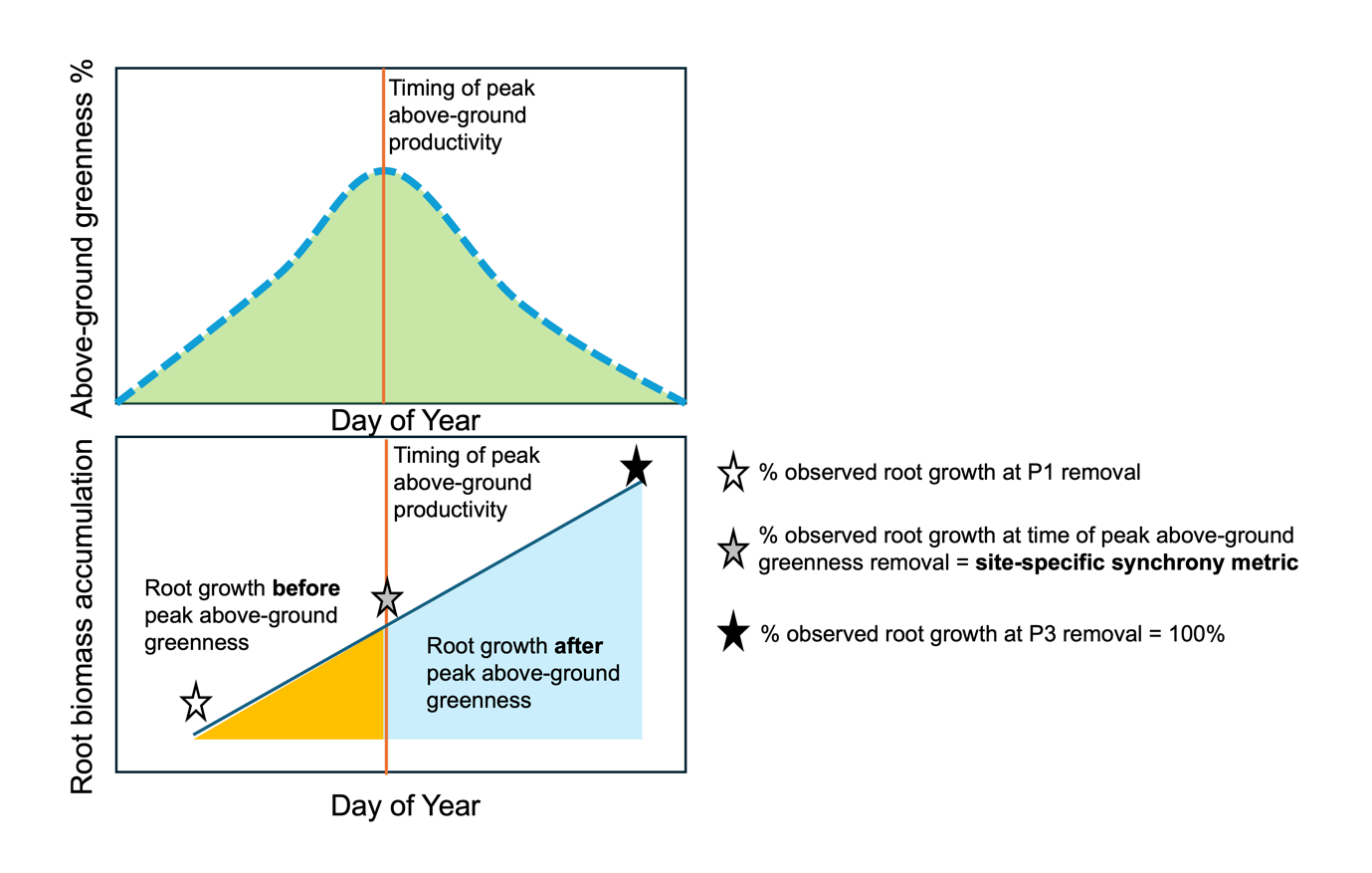


**Figure S1.** Schematic of the site-specific “synchrony metric” described in the methods and calculated using **Equation 3**.


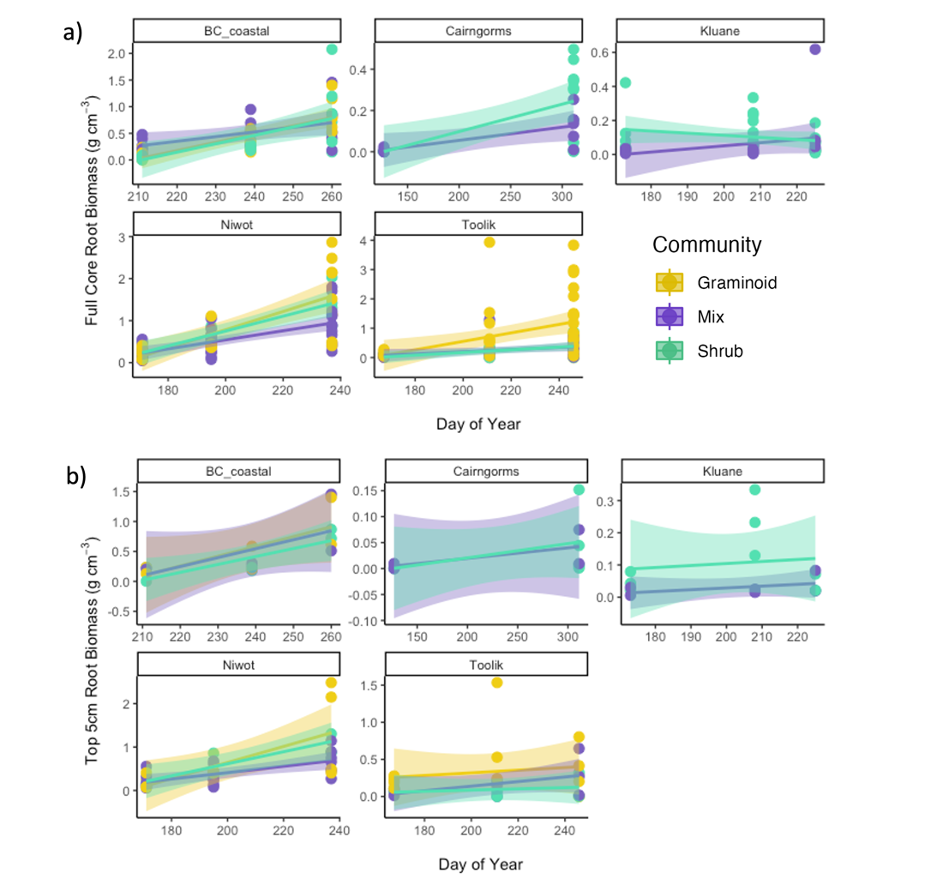


**Figure S2.** While relative magnitudes of root biomass differ across both data types, the differences between community types at each site remain consistent. Root Biomass accumulation over time categorised by plant community type. Panel (a) includes data calculated from the full length of each core. Panel (b) includes data calculated from only the top 5cm of each core.


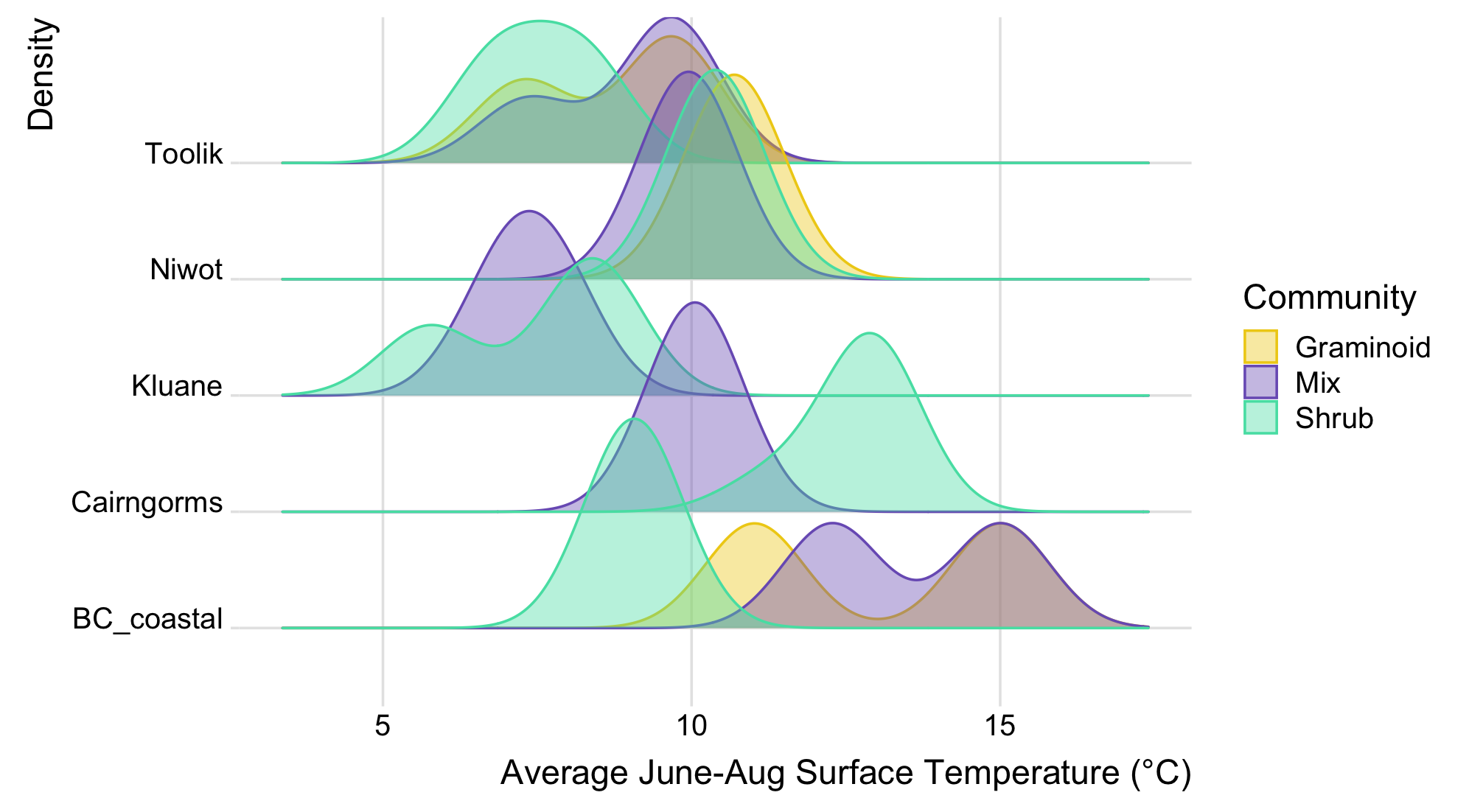


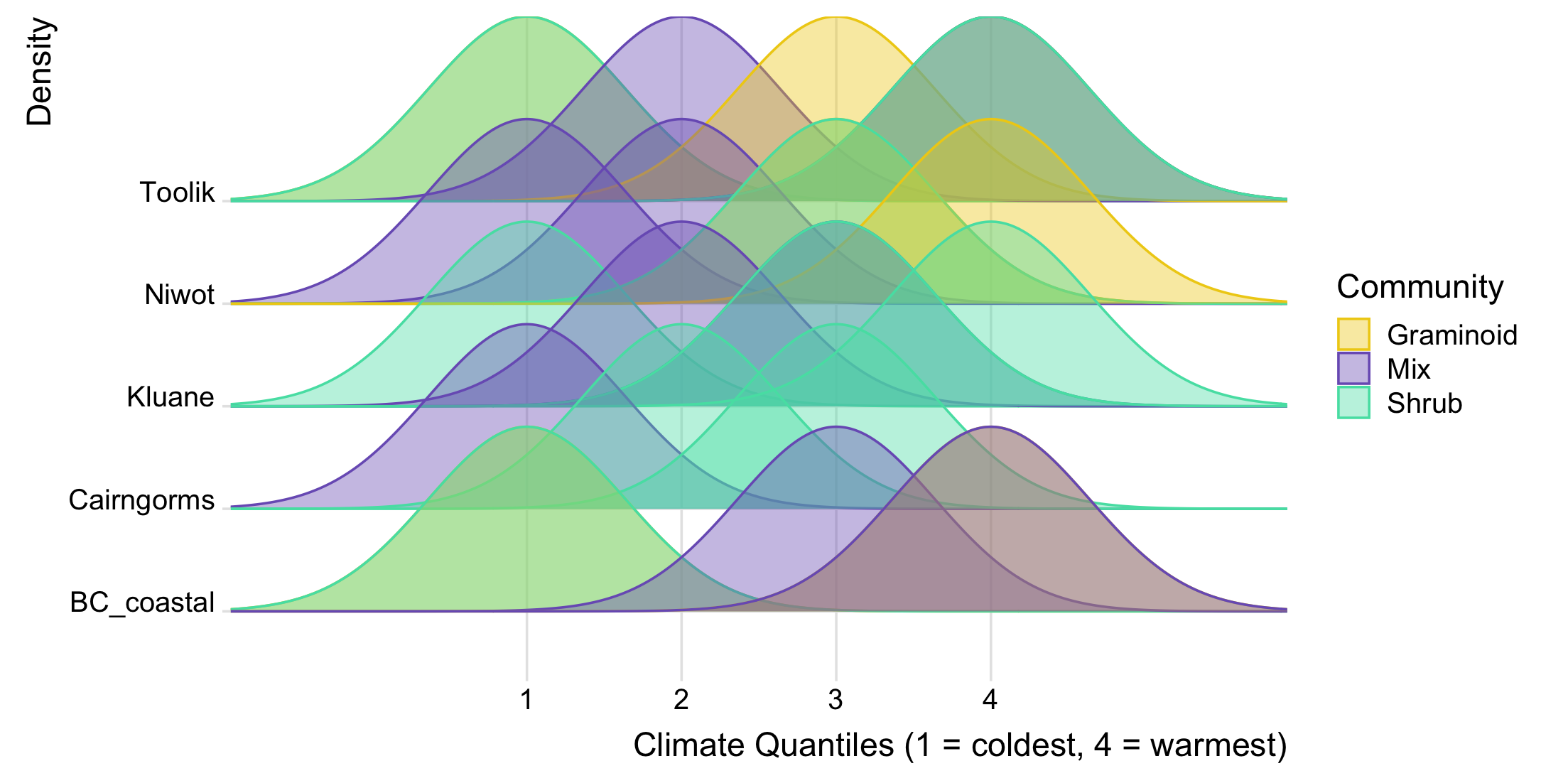


**Figure S3.** Community type and surface temperature do not covary across the sites. Distribution of summer surface temperatures by site, coloured by community type. In the top panel, climate is represented by average July-August surface temperature. In the bottom panel, climate is represented by climate quantile classifications.


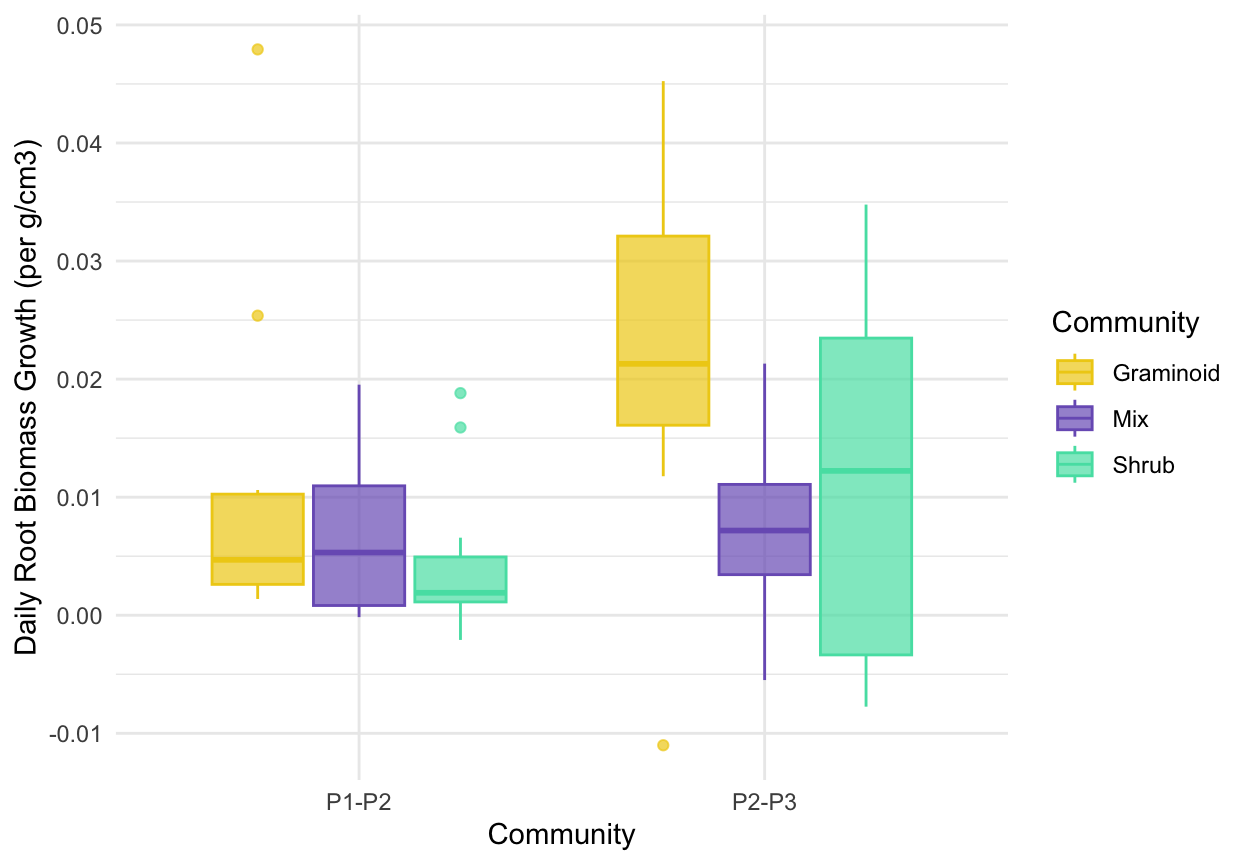


**Figure S4:** Root growth rate accelerates across all community sites, but especially in graminoid-dominated plots. Daily root growth rates between P1 and P2, and daily root growth rates between P2 and P3 across sites, coloured by community type.

**Table S2:** Statistical results for the hierarchical Bayesian models relating local surface temperature variation (i.e., climate quantiles 1-4), community plant type (graminoid, shrub, mix), and phenophase timing (P1, P2, P3, biomass model only) to root biomass, daily root growth rates, and zero-centered above-vs below-ground asynchrony. These models included ‘Site’ as a random intercept and an interactive term between community plant type and phenophase timing.

| Model name | Term | Estimate | Std. error | Lower 95% CI | Upper 95% CI |
| --- | --- | --- | --- | --- | --- |
| Root Biomass versus Temperature and Community and Phenophase | Intercept | 0.16 | 0.16 | -0.17 | 0.47 |
|  | CommunityMix | -0.04 | 0.1 | -0.23 | 0.18 |
|  | CommunityShrub | -0.01 | 0.11 | -0.22 | 0.22 |
|  | Core_IDP2 | 0.21 | 0.09 | 0.04 | 0.4 |
|  | Core_IDP3 | 0.89 | 0.09 | 0.73 | 1.07 |
|  | quantilegroup2 | 0.12 | 0.08 | -0.03 | 0.27 |
|  | quantilegroup3 | 0.11 | 0.07 | -0.02 | 0.25 |
|  | quantilegroup4 | 0.02 | 0.05 | -0.08 | 0.13 |
|  | CommunityMix:Core_IDP2 | -0.01 | 0.13 | -0.26 | 0.23 |
|  | CommunityShrub:Core_IDP2 | -0.03 | 0.14 | -0.3 | 0.24 |
|  | CommunityMix:Core_IDP3 | -0.53 | 0.13 | -0.79 | -0.29 |
|  | CommunityShrub:Core_IDP3 | -0.55 | 0.14 | -0.83 | -0.27 |
|  | Site__Intercept | 0.27 | 0.14 | 0.11 | 0.66 |
|  | sigma | 0.28 | 0.02 | 0.23 | 0.32 |
|  | alpha | 7.8 | 2.14 | 4.21 | 12.68 |
| Root Growth Rate versus Temperature and Community | Intercept | 0.01 | 0.01 | 0 | 0.02 |
|  | Community: Mix | -0.01 | 0 | -0.01 | -0.01 |
|  | Community: Shrub | -0.01 | 0 | -0.01 | -0.00 |
|  | Climate Quantile 2 | 0 | 0 | 0 | 0.01 |
|  | Climate Quantile 3 | 0 | 0 | 0 | 0.01 |
|  | Climate Quantile 4 | 0 | 0 | -0.01 | 0 |
|  | Site__Intercept | 0.01 | 0.01 | 0 | 0.02 |
|  | sigma | 0 | 0 | 0 | 0.01 |
|  | Site[BC_coastal,Intercept] | 0 | 0 | 0 | 0.01 |
|  | Site[Cairngorms,Intercept] | 0 | 0 | -0.02 | 0 |
|  | Site[Kluane,Intercept] | -0.01 | 0 | -0.02 | 0 |
|  | Site[Niwot,Intercept] | 0.01 | 0 | 0 | 0.02 |
|  | Site[Toolik,Intercept] | 0 | 0 | -0.01 | 0.01 |
| Root Synchrony Metric versus Temperature and Community | Intercept | 4.63 | 1.94 | 0.81 | 8.55 |
|  | Community: Mix | -7.59 | 1.86 | -11.22 | -3.87 |
|  | Community: Shrub | 0.21 | 1.91 | -3.54 | 3.94 |
|  | Climate Quantile 2 | 4.07 | 2.6 | -1.1 | 9.25 |
|  | Climate Quantile 3 | -4.38 | 1.95 | -8.28 | -0.5 |
|  | Climate Quantile 4 | -3.24 | 1.85 | -6.88 | 0.48 |
|  | Site__Intercept | 1 | 0.89 | 0.03 | 3.33 |
|  | sigma | 3.9 | 0.53 | 3.01 | 5.11 |
|  | Site[BC_coastal,Intercept] | -0.3 | 1 | -2.83 | 1.44 |
|  | Site[Cairngorms,Intercept] | 0.4 | 1.11 | -1.41 | 3.25 |
|  | Site[Kluane,Intercept] | -0.03 | 0.97 | -2.17 | 2 |
|  | Site[Niwot,Intercept] | 0 | 0.87 | -1.93 | 1.9 |
|  | Site[Toolik,Intercept] | -0.14 | 0.92 | -2.36 | 1.69 |

**Table S3:** Statistical results for the hierarchical Bayesian models relating local surface temperature variation (i.e., climate quantiles 1-4), and community type (graminoid, shrub, mix), and phenophase timing (P1, P2, P3 -biomass model only) to root biomass, daily root growth rates, and above-vs below-ground asynchrony. These models included ‘Site’ as a random intercept. These results only include root biomass data from the top 5cm of each core.

| Model Name | Term | Estimate | Std. Error | Lower 95% CI | Upper 95% CI |
| --- | --- | --- | --- | --- | --- |
| Root Biomass versus Temperature and Community and Phenophase | Intercept | 0.3 | 0.17 | -0.07 | 0.64 |
|  | Community: Mix | -0.12 | 0.07 | -0.25 | 0.01 |
|  | Community: Shrub | -0.09 | 0.07 | -0.23 | 0.06 |
|  | Core_ID:P2 | 0.15 | 0.06 | 0.03 | 0.26 |
|  | Core_ID:P3 | 0.31 | 0.06 | 0.19 | 0.43 |
|  | Climate Quantile 2 | 0.07 | 0.09 | -0.1 | 0.24 |
|  | Climate Quantile 3 | 0.08 | 0.07 | -0.06 | 0.22 |
|  | Climate Quantile 4 | 0.07 | 0.07 | -0.07 | 0.2 |
|  | Site_Intercept | 0.31 | 0.16 | 0.12 | 0.74 |
|  | sigma | 0.25 | 0.02 | 0.21 | 0.29 |
|  | rSite[BCcoastal,Intercept] | 0.2 | 0.16 | -0.1 | 0.55 |
|  | rSite[Cairngorms,Intercept] | -0.21 | 0.18 | -0.58 | 0.13 |
|  | rSite[Kluane,Intercept] | -0.16 | 0.16 | -0.51 | 0.17 |
|  | rSite[Niwot,Intercept] | 0.22 | 0.16 | -0.1 | 0.56 |
|  | rSite[Toolik,Intercept] | -0.06 | 0.16 | -0.39 | 0.28 |
| Root Growth Rate versus Temperature and Community | Intercept | 0.01 | 0.01 | -0.01 | 0.02 |
|  | Community: Mix | 0 | 0 | -0.01 | 0 |
|  | Community: Shrub | 0 | 0 | -0.01 | 0 |
|  | Climate Quantile 2 | 0 | 0.01 | -0.01 | 0.01 |
|  | Climate Quantile 3 | 0.01 | 0 | 0 | 0.01 |
|  | Climate Quantile 4 | 0 | 0 | -0.01 | 0.01 |
|  | Site_Intercept | 0.01 | 0.01 | 0 | 0.03 |
|  | sigma | 0.01 | 0 | 0.01 | 0.01 |
|  | rSite[BCcoastal,Intercept] | 0.01 | 0.01 | 0 | 0.02 |
|  | rSite[Cairngorms,Intercept] | -0.01 | 0.01 | -0.02 | 0.01 |
|  | rSite[Kluane,Intercept] | 0 | 0.01 | -0.02 | 0.01 |
|  | rSite[Niwot,Intercept] | 0.01 | 0.01 | -0.01 | 0.02 |
|  | rSite[Toolik,Intercept] | 0 | 0.01 | -0.02 | 0.01 |
| Root Synchrony Metric versus Temperature & Community | Intercept | 4.63 | 1.89 | 0.83 | 8.3 |
|  | Community: Mix | -7.61 | 1.88 | -11.31 | -3.83 |
|  | Community: Shrub | 0.25 | 1.9 | -3.49 | 4.05 |
|  | Climate Quantile 2 | 4.08 | 2.61 | -1.1 | 9.16 |
|  | Climate Quantile 3 | -4.43 | 1.91 | -8.09 | -0.68 |
|  | Climate Quantile 4 | -3.28 | 1.84 | -6.83 | 0.33 |
|  | Site_Intercept | 1.06 | 1.03 | 0.03 | 3.37 |
|  | sigma | 3.9 | 0.54 | 3.01 | 5.13 |
|  | rSite[BCcoastal,Intercept] | -0.31 | 0.99 | -2.75 | 1.41 |
|  | rSite[Cairngorms,Intercept] | 0.45 | 1.18 | -1.48 | 3.5 |
|  | rSite[Kluane,Intercept] | -0.03 | 1 | -2.2 | 2.1 |
|  | rSite[Toolik,Intercept] | 0.01 | 0.9 | -1.91 | 1.97 |

**Table S4:** Statistical results for the hierarchical Bayesian models relating local surface temperature continuous data, community plant type (graminoid, shrub, mix), and phenophase timing (P1, P2, P3, biomass model only) to root biomass, daily root growth rates, and zero-centered above-vs below-ground asynchrony. These models included ‘Site’ as a random intercept and an interactive term between community plant type and phenophase timing.

| Model name | Term | Estimate | Std. error | Lower 95% CI | Upper 95% CI |
| --- | --- | --- | --- | --- | --- |
| Root Biomass versus Temperature and Community and Phenophase | Intercept | 0.21 | 0.13 | -0.08 | 0.46 |
|  | CommunityMix | 0.01 | 0.09 | -0.17 | 0.2 |
|  | CommunityShrub | 0.04 | 0.1 | -0.17 | 0.24 |
|  | Core_IDP2 | 0.22 | 0.09 | 0.05 | 0.4 |
|  | Core_IDP3 | 0.88 | 0.09 | 0.72 | 1.08 |
|  | Daily summer surface temp | 0 | 0.02 | -0.03 | 0.03 |
|  | CommunityMix:Core_IDP2 | -0.06 | 0.12 | -0.3 | 0.17 |
|  | CommunityShrub:Core_IDP2 | -0.08 | 0.13 | -0.35 | 0.19 |
|  | CommunityMix:Core_IDP3 | -0.56 | 0.12 | -0.8 | -0.33 |
|  | CommunityShrub:Core_IDP3 | -0.61 | 0.14 | -0.89 | -0.33 |
|  | Site__Intercept | 0.23 | 0.12 | 0.09 | 0.55 |
|  | sigma | 0.28 | 0.02 | 0.24 | 0.33 |
|  | alpha | 7.88 | 2.24 | 4.14 | 12.72 |
|  | Site[BC_coastal,Intercept] | 0.06 | 0.12 | -0.18 | 0.32 |
|  | Site[Cairngorms,Intercept] | -0.05 | 0.13 | -0.32 | 0.22 |
|  | Site[Kluane,Intercept] | -0.19 | 0.12 | -0.46 | 0.06 |
|  | Site[Niwot,Intercept] | 0.21 | 0.12 | -0.01 | 0.47 |
|  | Site[Toolik,Intercept] | -0.01 | 0.12 | -0.24 | 0.25 |
| Root Growth Rate versus Temperature and Community | Intercept | 0.01 | 0 | 0.01 | 0.02 |
|  | CommunityMix | -0.01 | 0 | -0.01 | -0.01 |
|  | CommunityShrub | -0.01 | 0 | -0.01 | 0 |
|  | Daily summer surface temp | 0 | 0 | 0 | 0 |
|  | Site__Intercept | 0.01 | 0 | 0 | 0.02 |
|  | sigma | 0 | 0 | 0 | 0.01 |
|  | Site[BC_coastal,Intercept] | 0 | 0 | -0.01 | 0.01 |
|  | Site[Cairngorms,Intercept] | 0 | 0 | -0.01 | 0 |
|  | Site[Kluane,Intercept] | -0.01 | 0 | -0.02 | 0 |
|  | Site[Niwot,Intercept] | 0.01 | 0 | 0 | 0.01 |
|  | Site[Toolik,Intercept] | 0 | 0 | -0.01 | 0.01 |
| Root Synchrony Metric versus Temperature and Community | Intercept | 2.14 | 1.46 | -0.78 | 5.04 |
|  | CommunityMix | -5.27 | 1.78 | -8.77 | -1.76 |
|  | CommunityShrub | -0.47 | 1.97 | -4.31 | 3.38 |
|  | Daily summer surface temp | -1.18 | 0.61 | -2.42 | 0 |
|  | Site__Intercept | 1.11 | 0.99 | 0.03 | 3.77 |
|  | sigma | 4.29 | 0.57 | 3.34 | 5.59 |
|  | Site[BC_coastal,Intercept] | -0.43 | 1.1 | -3.2 | 1.4 |
|  | Site[Cairngorms,Intercept] | 0.32 | 1.19 | -1.84 | 3.21 |
|  | Site[Kluane,Intercept] | 0.09 | 1.06 | -2.17 | 2.51 |
|  | Site[Niwot,Intercept] | 0.21 | 0.98 | -1.74 | 2.45 |
|  | Site[Toolik,Intercept] | -0.22 | 1 | -2.5 | 1.76 |

**Table S5:** Statistical results for the hierarchical Bayesian models relating local surface temperature continuous data, community type (graminoid, shrub, mix), and phenophase timing (P1, P2, P3 -biomass model only) to root biomass, daily root growth rates, and above-vs below-ground asynchrony. These models included ‘Site’ as a random intercept. These results only include root biomass data from the top 5cm of each core.

| Model name | Term | Estimate | Std. error | | Lower 95% CI | Upper 95% CI |
| --- | --- | --- | --- | --- | --- | --- |
| Root Biomass versus Temperature and Community and Phenophase | Intercept | 0.24 | 0.14 | | -0.05 | 0.49 |
|  | CommunityMix | 0 | 0.1 | | -0.2 | 0.2 |
|  | CommunityShrub | 0.03 | 0.11 | | -0.21 | 0.24 |
|  | Core_IDP2 | 0.19 | 0.11 | | -0.03 | 0.4 |
|  | Core_IDP3 | 0.28 | 0.11 | | 0.07 | 0.5 |
|  | Daily summer surface temp | 0.02 | 0.02 | | -0.03 | 0.06 |
|  | CommunityMix:Core_IDP2 | -0.14 | 0.14 | | -0.42 | 0.14 |
|  | CommunityShrub:Core_IDP2 | -0.09 | 0.16 | | -0.4 | 0.23 |
|  | CommunityMix:Core_IDP3 | -0.06 | 0.14 | | -0.34 | 0.21 |
|  | CommunityShrub:Core_IDP3 | -0.13 | 0.15 | | -0.43 | 0.17 |
|  | Site__Intercept | 0.21 | 0.13 | | 0.07 | 0.55 |
|  | sigma | 0.32 | 0.03 | | 0.27 | 0.38 |
|  | alpha | 7.53 | 2.21 | | 3.9 | 12.5 |
|  | Site[BC_coastal,Intercept] | 0.12 | 0.12 | | -0.1 | 0.38 |
|  | Site[Cairngorms,Intercept] | -0.09 | 0.13 | | -0.37 | 0.16 |
|  | Site[Kluane,Intercept] | -0.11 | | 0.12 | -0.36 | 0.12 |
|  | Site[Niwot,Intercept] | 0.16 | 0.11 | | -0.06 | 0.4 |
|  | Site[Toolik,Intercept] | -0.09 | 0.11 | | -0.33 | 0.15 |
| Root Growth Rate versus Temperature and Community | Intercept | 0.01 | 0.01 | | 0 | 0.02 |
|  | CommunityMix | 0 | 0 | | -0.01 | 0 |
|  | CommunityShrub | 0 | 0 | | -0.01 | 0 |
|  | Daily summer surface temp | 0 | 0 | | 0 | 0 |
|  | Site__Intercept | 0.01 | 0.01 | | 0 | 0.02 |
|  | sigma | 0.01 | 0 | | 0.01 | 0.01 |
|  | Site[BC_coastal,Intercept] | 0.01 | 0.01 | | 0 | 0.02 |
|  | Site[Cairngorms,Intercept] | 0 | 0.01 | | -0.02 | 0.01 |
|  | Site[Kluane,Intercept] | 0 | 0.01 | | -0.02 | 0.01 |
|  | Site[Niwot,Intercept] | 0.01 | 0.01 | | 0 | 0.02 |
|  | Site[Toolik,Intercept] | 0.01 | 0.01 | | 0 | 0.02 |
| Root Synchrony Metric versus Temperature and Community | Intercept | 2.19 | 1.49 | | -0.66 | 5.2 |
|  | CommunityMix | -5.27 | 1.83 | | -8.95 | -1.69 |
|  | CommunityShrub | -0.5 | 2 | | -4.48 | 3.44 |
|  | Daily summer surface temp | -1.18 | 0.61 | | -2.42 | 0.01 |
|  | Site__Intercept | 1.13 | 1.02 | | 0.04 | 3.81 |
|  | sigma | 4.31 | 0.57 | | 3.37 | 5.61 |
|  | Site[BC_coastal,Intercept] | -0.42 | 1.14 | | -3.28 | 1.5 |
|  | Site[Cairngorms,Intercept] | 0.3 | 1.18 | | -1.8 | 3.27 |
|  | Site[Kluane,Intercept] | 0.07 | 1.09 | | -2.23 | 2.5 |
|  | Site[Niwot,Intercept] | 0.19 | 0.96 | | -1.74 | 2.34 |
|  | Site[Toolik,Intercept] | -0.28 | 1.03 | | -2.82 | 1.63 |
